# Directional Muscle-to-Bone Crosstalk via the Muscle Secretome: A Systematic Review

**DOI:** 10.3390/ijms27209102

**Published:** 2026-10-14

**Authors:** Žana Rus, Simona Jurković Mlakar, Barbara Ostanek, Janja Marc

**Affiliations:** 1 Department of Clinical Biochemistry, Faculty of Pharmacy, University of Ljubljana, 1000 Ljubljana, Slovenia; zana.rus@ffa.uni-lj.si (Ž.R.); simona.jurkovicmlakar@ffa.uni-lj.si (S.J.M.); barbara.ostanek@ffa.uni-lj.si (B.O.); 2 Clinical Institute of Clinical Chemistry and Biochemistry, University Clinical Center Ljubljana, 1000 Ljubljana, Slovenia

**Keywords:** conditioned medium, extracellular vesicles, mesenchymal stem/stromal cells, osteoblasts, osteoclasts, osteosarcopenia

## Abstract

Skeletal muscle communicates biochemically with bone via a secretome consisting of soluble factors and extracellular vesicles (EVs). This secretory activity is regulated by mechanical stimuli and the muscle’s metabolic state. The aim of this systematic review was to evaluate the effects of the muscle secretome, specifically muscle-derived conditioned media and EVs, on bone mesenchymal stem/stromal cells (MSCs), osteoblasts, osteoclasts, and bone-related outcomes in in vitro and in vivo models. Following PRISMA guidelines, PubMed and Web of Science were searched for original studies testing the muscle-derived secretome on MSCs, bone cells, or bone tissue, identifying 572 studies, of which 31 met the inclusion criteria. Risk of bias was assessed using SYRCLE for in vivo studies and a modified SYRCLE tool for in vitro studies. MISEV2023 terminology was applied. The majority of the included studies reported predominantly positive effects of secretome from healthy or mechanically stimulated muscle on MSC proliferation and osteogenic differentiation, improved osteoblast function, and suppressed osteoclast formation, with multiple in vivo confirmations. Conversely, secretome from atrophic/estrogen-deficient muscle inhibited osteogenesis and promoted osteoclastogenesis. Risk of bias and reporting limitations were common: heterogeneous dosing/reporting, incomplete reporting of purity, contaminants, and functional assay controls according to MISEV guidelines, and suboptimal SYRCLE domains in in vivo studies. The direction of muscle-to-bone biochemical crosstalk reflects muscle health—anabolic/antiresorptive when muscle is healthy or mechanically active, catabolic/proresorptive when muscle is atrophic or estrogen-deficient. These findings support exercise- and exerkine-based approaches, as well as EV-based therapeutics for osteoporosis or osteosarcopenia.

## 1. Introduction

Osteoporosis and sarcopenia frequently co-occur in older adults, culminating in osteosarcopenia, a condition associated with greater fracture risk, disability, and mortality. The burden rises with population aging, especially post-menopause [1,2]. With global population aging, the prevalence of osteosarcopenia is projected to rise dramatically, affecting up to two billion older adults by 2050 [3]. Impaired biochemical crosstalk between muscle and bone is considered a core pathogenic mechanism. Understanding the molecular architecture of this communication is thus critical for identifying preventive and therapeutic strategies [2].

At the physiological level, these disorders reflect a breakdown of the integrated musculoskeletal unit, where bone and muscle communicate through mechanical and biochemical pathways. Muscle contractions generate mechanical forces that stimulate bone formation, regulate bone mineral density (BMD) and maintain skeletal strength. Beyond postural stabilization and structural integrity of the human body, skeletal muscle functions as an endocrine organ by secreting signaling molecules—collectively termed myokines—including cytokines, growth factors, and hormones. These molecules, present in the muscle secretome, exert autocrine, paracrine, and endocrine effects that modulate bone cell fate and remodeling. The composition of the secretome varies depending on physiological and pathological states, such as health, aging, exercise, and muscle atrophy [4,5,6,7].

Among the first identified myokines were myostatin and interleukin 6 (IL-6) [8,9,10], both of which have profound effects on bone metabolism. Myostatin, for example, inhibits osteoblast differentiation and promotes osteoclastogenesis, thereby stimulating bone resorption; IL-6 also indirectly influences bone resorption by promoting osteoclast formation through osteoblastic/stromal cell production of receptor activator of nuclear kappa-B ligand (RANKL). The interplay between muscle-derived factors and bone cells is complex and context-dependent, influenced by mechanical stimuli, hormonal status, and disease conditions [8,9,11,12,13,14].

Recent research has expanded this paradigm by highlighting that the secretome comprises not only proteins but also extracellular vesicles (EVs), cell-derived vesicles enclosed by a lipid bilayer. They are commonly classified according to their biogenesis into exosomes, microvesicles, and apoptotic bodies. The terms have often been used interchangeably due to the lack of a unified characterization of vesicles. Therefore, throughout the text, we use the term “extracellular vesicles” for all vesicle types. EVs mediate intercellular communication by transporting bioactive cargo, such as proteins, lipids, and RNAs (including mRNA, microRNA (miRNA), and other non-coding RNAs), and affect both recipient and donor cells [15,16]. miRNAs implicated in muscle–bone crosstalk include miR-27a-3p, miR-34a [17], miR-92a-1-5p [18], miR-126a-3p, miR-450b-5p, miR-669a-5p, miR-5100 [19] and miR-196a-5p [20]. These molecules modulate osteogenic differentiation, osteoclastogenesis, and signaling pathways such as miR-92a-3p/PTEN/Akt (miR-92a-3p/phosphatase and tensin homolog/protein kinase B) [16], β-catenin [21,22], and Hippo [23], highlighting their potential as therapeutic targets.

Although prior reviews have emphasized individual myokines (e.g., irisin, myostatin, fibroblast growth factor 2 (FGF-2), IL-6, IL-15 [24]), this approach underestimates the concerted, co-delivered nature of the secretome that bone cells experience in physiological contexts. The present work instead focuses on the whole muscle-derived secretome, including conditioned medium (CM) and EVs. Given the importance of consistent terminology, we therefore follow the Minimal Information for Studies of Extracellular Vesicles 2023 (MISEV2023) and use “EVs” as the default term [25].

Despite the rapidly growing literature on muscle–bone crosstalk, there has been no systematic review conducted and reported in accordance with the Preferred Reporting Items for Systematic Reviews and Meta-Analyses (PRISMA) guidelines that (i) aggregates CM and EV effects on mesenchymal stem/stromal cells (MSCs) [26], osteoblasts (OBs), osteoclasts (OCs), and bone outcomes across in vitro and in vivo models; (ii) disentangles muscle-state-dependent directionality (healthy/mechanically stimulated vs. atrophic/estrogen-deficient/inflamed); (iii) critically appraises risk of bias (RoB) and reporting quality, including MISEV compliance; and (iv) maps recurrent molecular pathways/cargo. Addressing these points is directly relevant to osteosarcopenia and could inform exercise/exerkine strategies and EV-centric therapeutics.

We conducted a systematic review to:

1. Synthesize the effects of muscle-derived CM and EVs on MSCs, OBs, OCs, and bone outcomes across in vitro and in vivo studies;
2. Stratify findings by muscle condition (healthy/mechanically stimulated vs. atrophic/estrogen-deficient/inflamed);
3. Evaluate RoB and reporting quality using the Systematic Review Center for Laboratory Animal Experimentation (SYRCLE) and a modified SYRCLE for in vitro studies, and assess adherence to MISEV2023;
4. Summarize mechanistic mediators (e.g., miRNAs, signaling pathways, metabolic cargo);
5. Identify gaps that must be bridged to enable clinical translation.

## 2. Materials and Methods

Two protocols, one for human studies and one for animal studies, were registered on PROSPERO (CRD420261306525 and CRD420261308106). This systematic review was conducted in accordance with the PRISMA guidelines [27,28]. The PRISMA checklist is presented in Supplementary Table S1.

### 2.1. Search Strategy

Two electronic databases (PubMed and Web of Science) were searched on 6 January 2026 by two independent pairs of researchers using predefined PICO (Population, Intervention, Comparison, Outcome) terms (Supplementary Table S2). Full Boolean strategies are provided in Supplementary Table S3. Searches were limited to English and ≥2016 to reflect contemporary EV and secretome reporting standards.

### 2.2. Eligibility Criteria

The inclusion criteria were as follows: (1) original research, (2) studies conducted in human, animal, or cellular models, (3) evaluating the effect of muscle-derived secretome, CM, EVs, or exosomes on MSCs, bone cells, or bone tissue, (4) publications in English, (5) publications from 2016 onwards.

The exclusion criteria were as follows: (1) full text not accessible, (2) non-original research (e.g., reviews, editorials), (3) book chapters, (4) retracted publications, and (5) conference proceedings.

### 2.3. Study Selection

**Figure 1:**
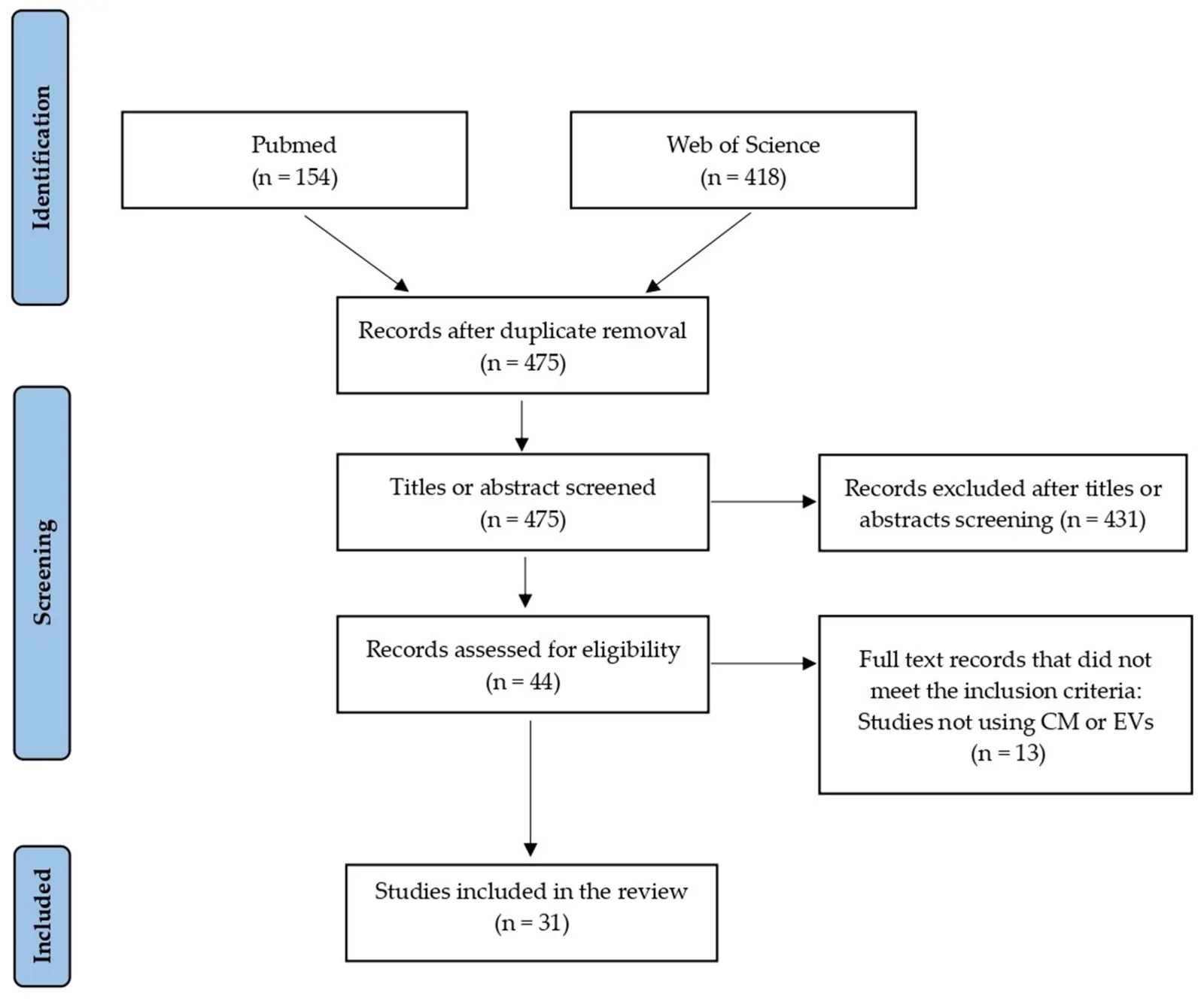
Flowchart of articles included in the systematic review.

Records were imported and deduplicated using the Automated Systematic Search Deduplicator (ASySD). Titles and abstracts were screened independently by two reviewers (from a pool of four). All disagreements were resolved through discussion; if consensus could not be reached, a third reviewer was consulted. Full-text articles that passed the screening were then assessed for eligibility. A PRISMA flow diagram summarizes the study selection (Figure 1).

### 2.4. Data Extraction

Data were extracted using standardized forms for in vivo and in vitro studies to capture: study identifiers (authors and year), donor muscle source (species/cell line; healthy/injured/mechanically stimulated), intervention type (CM, EVs, author-reported “exosomes”), target animal(s) or cell type(s), reported mechanisms (signaling pathway, miRNA, proteins, metabolic cargo), and main outcomes (primary and secondary). This structured approach ensured consistency and facilitated comparative analysis across studies.

### 2.5. Types of Outcome Measurements

The outcomes of interest were categorized into two groups: primary and secondary. These were defined based on the objective of the systematic review, which was to assess the effects of muscle secretome on MSCs, OBs, OCs, and other bone-associated outcomes, as well as to summarize mechanistic mediators.

#### 2.5.1. Primary Outcomes

Primary outcomes were further stratified into in vitro and in vivo experiments. In vitro experiments focused on bone-related cellular processes, while in vivo experiments assessed bone parameters.

In vitro: osteogenesis (e.g., alkaline phosphatase (ALP) activity, alizarin red S (ARS)/von Kossa staining), osteoclastogenesis (tartrate-resistant acid phosphatase (TRAP) staining, resorption assays), OB/OC gene/protein expression, and cell proliferation/migration/viability.

In vivo: bone microarchitecture (micro-computed tomography (micro-CT), histomorphometry), bone turnover markers, and bone mass loss/protection from bone loss in disease models (e.g., ovariectomy (OVX), glucocorticoids, disuse/denervation, diabetes).

#### 2.5.2. Secondary Outcomes

Secondary outcomes included mechanistic mediators, such as miR-92a-3p/PTEN/AKT, β-catenin, PI3K (phosphatidylinositol 3-kinase)/AKT, YAP (yes-associated protein)/Hippo pathway, lactate dehydrogenase A (LDHA)-linked metabolic reprogramming, and inhibitory signals such as galectin-3 or tumor necrosis factor-α (TNF-α)-modified cargo.

### 2.6. Risk of Bias Assessment

We assessed in vivo studies using SYRCLE’s Risk of Bias (RoB) tool [29] and in vitro studies using a modified SYRCLE’s RoB tool tailored to cell-based experiments [30]. Ratings were performed by one reviewer and verified by a second, with domain- and study-level summaries reported (Supplementary Tables S4 and S5).

### 2.7. Terminology and Reporting Standards (MISEV2023)

We followed the MISEV2023 guidelines [25], using “EVs” as the default term and reserving “exosomes” only when biogenesis-specific evidence was provided in the primary study. We note that many isolates may contain non-vesicular extracellular particles; where authors used “exosomes” without biogenesis proof, we reclassified them as EVs and annotated this in tables or footnotes. We also highlight characterization gaps (e.g., marker panels, contaminants, particle-to-protein ratios) and dosing normalization differences (particle count, protein mass, volume).

### 2.8. Synthesis Methods

Given the heterogeneity in models, interventions, dosing, and outcomes, we conducted a narrative synthesis stratified by muscle state (healthy/mechanically stimulated vs. atrophic/estrogen-deficient/inflamed) and by target cell type (MSCs, OBs, OCs), with cross-referencing to in vivo bone outcomes. Mechanistic signals were mapped across studies to identify recurrent axes and points of convergence/divergence. Where feasible, directionality (anabolic/antiresorptive vs. catabolic/proresorptive) was summarized in tabular form ().

The substantial heterogeneity in models, interventions, dosing, outcome measurements, and reporting formats precluded a quantitative synthesis. Therefore, studies were qualitatively classified as exhibiting predominantly anabolic/antiresorptive or catabolic/proresorptive effects based on the overall direction of the reported target cell type or bone-related outcomes. When multiple outcomes were reported, classification was based on the predominant biological effect observed across osteogenic differentiation, osteoblast function (activity, migration, proliferation, ferroptosis), osteoclast formation, and bone mass or bone loss endpoints.

## 3. Results

### 3.1. Literature Search Results

The database search retrieved 572 records (PubMed: 154; Web of Science: 418). After automated deduplication (n = 97) using ASySD, 475 unique records remained. Screening of titles and abstracts excluded 431 items, primarily reviews, non-original work or studies not examining muscle-derived CM/EVs on bone-related targets. The remaining 44 articles underwent full-text assessment, resulting in the exclusion of 13 articles that did not meet the eligibility criteria, specifically because they did not use muscle-derived CM or EVs. The excluded articles are listed in Supplementary Table S6. The final synthesis included 31 studies (Figure 1).

### 3.2. Characteristics of Included Studies

The 31 included studies comprised 18 in vivo and 31 in vitro experiments, with several integrating both approaches. Donor muscle sources included C2C12 mouse myoblasts (the majority), L6 rat myoblasts, human skeletal muscle tissue or myotubes, human aortic smooth muscle cells, and various animal skeletal muscles. Interventions included bone cell treatments with CM (n = 12), EV isolates (n = 8), “exosomes” (n = 9, reclassified as EVs under MISEV2023 due to a lack of demonstrated biogenesis), and two studies assessing both CM and EVs. Target bone cell types included MSCs, OBs, OCs, osteocyte-like cells, and mixed bone marrow cultures; in vivo models involved bone assessment in mice and rats. The main characteristics of the selected studies are summarized in Supplementary Table S7.

Outcomes were assessed using both in vitro and in vivo approaches. In vitro analyses comprised ALP activity, ARS/von Kossa staining, OB/OC gene and protein expression, cell proliferation and migration assays, and TRAP staining. In vivo outcomes were evaluated using micro-CT, histomorphometry, and bone turnover markers. Pathological muscle states (e.g., atrophy, estrogen deficiency, inflammation, denervation, diabetes) were modeled to test secretome directionality.

### 3.3. Quality Assessment of Selected Studies

SYRCLE was used for in vivo studies and a modified SYRCLE tool for in vitro studies to assess six types of bias. Both tools assessed six bias domains, although the distribution of the individual domains differed between the two versions. In the SYRCLE tool for assessing RoB in in vivo studies, domains 1–3 corresponded to selection bias, domains 4–5 to performance bias, domains 6–7 to detection bias, domain 8 to attrition bias, domain 9 to reporting bias, and domain 10 to other sources of bias. In the modified SYRCLE RoB tool, domains 1–2 corresponded to selection bias, domains 3–4 to performance bias, and domains 5–7 to detection bias, while domains 8, 9, and 10 corresponded to attrition bias, reporting bias, and other sources of bias, respectively, as in the SYRCLE tool for assessing RoB in in vivo studies. Because the domains and their distribution across bias categories differ between the two tools, the RoB assessments of in vitro and in vivo studies were not directly or fully comparable.

#### 3.3.1. In Vivo Studies

Most in vivo studies showed unclear or high risk of bias across SYRCLE domains. Common deficiencies included insufficient reporting on allocation concealment, random housing, blinding, and random outcome assessment. Incomplete outcome data were the most frequent high-risk domain. Only a minority of studies described methods with sufficient rigor to meet low-risk criteria across multiple items (Table 1).

#### 3.3.2. In Vitro Studies

Reporting quality was generally stronger in in vitro studies. Baseline conditions, intervention descriptions, cell culture parameters, group comparisons, and outcome measurement methods were usually well documented. However, sample size calculations, blinding, and explicit handling of incomplete data were rarely reported (Table 2).

#### 3.3.3. Risk of Bias Patterns Across Included Studies

Overall patterns of risk of bias differed between in vitro and in vivo studies. Most in vivo studies were assessed as having a high risk of bias in the domain of incomplete outcome data, whereas selective outcome reporting and other sources of bias were generally reported appropriately. The risk of bias in other domains was frequently unclear, except for sequence generation, baseline characteristics, and blinding, which were more consistently reported.

For most in vitro studies, sufficient detail regarding baseline characteristics, interventions, culture conditions, group comparisons, outcome measurement, and selective outcome reporting was provided. However, ambiguity was frequently noted for the item addressing “other sources of bias”. In contrast, sample size calculation was commonly inadequately reported, while blinding and addressing incomplete outcome data were generally unclear.

### 3.4. Compliance with MISEV2023

EV characterization frequently fell short of MISEV2023 expectations, with overall low-to-moderate compliance (Supplementary Table S8). Isolation methods were generally well documented, and EV markers for validation, as well as detection and quantification methods, were commonly reported. In contrast, dosing units were more heterogeneous. Reporting of purity, contaminants, and functional assay controls was often inadequate or absent.

### 3.5. Effects of Muscle-Derived Secretome on Target Cell Type

#### 3.5.1. Effects on MSCs

Across models, CM and EVs from healthy or mechanically stimulated muscle consistently enhanced MSC proliferation and osteogenic differentiation [16,18,19,23,32,35,36,37,39,51,52]. Beyond identifying signaling pathways, several studies provided mechanistic evidence that muscle-derived EV cargo directly regulates MSC fate through coordinated effects on intracellular signaling, gene expression, and cellular metabolism. Mechanically stimulated myoblast-derived EVs were enriched in miR-92a-3p, which suppressed PTEN and consequently activated Akt signaling, resulting in enhanced MSC proliferation and osteogenic differentiation [16]. Similarly, myoblast-derived EVs containing Prrx2 (paired-related homeobox 2) promoted osteogenic differentiation through transcriptional activation of the long non-coding RNA MIR22HG (MIR22 host gene), which acted as a molecular sponge for miR-128 and increased YAP1 expression, thereby activating Hippo signaling and promoting osteogenic commitment of MSCs [23].

Other EV-associated miRNAs, including miR-873-3p, miR-5100, miR-126a-3p, miR-450b-5p, and miR-669-5p, were associated with increased expression of osteogenic markers and enhanced bone-forming activity, suggesting coordinated regulation of transcriptional programs required for osteoblast lineage commitment [19,39]. In addition to regulatory RNAs, EVs also transferred metabolic cargo. Muscle-derived EVs enriched with glycolytic enzymes induced metabolic reprogramming toward glycolysis in MSCs, increasing cellular bioenergetic capacity and supporting osteogenic differentiation [36].

Notably, the effects of EVs depended strongly on the physiological state of the donor muscle. While EVs from healthy or mechanically stimulated muscle promoted osteogenesis, EVs released from atrophic muscle exerted the opposite effect. Wang et al. demonstrated that atrophic muscle-derived EVs carried increased levels of miR-92a-1-5p, which inhibited osteogenic differentiation both in vitro and in vivo [18]. Similarly, Huang et al. reported that EVs from atrophic skeletal muscle impaired MSC osteogenic differentiation compared with EVs derived from healthy muscle tissue [35]. Collectively, these findings indicate that muscle-derived EVs function as biologically active carriers of regulatory RNAs and metabolic mediators that directly influence MSC proliferation, lineage commitment, and osteogenic differentiation, thereby translating muscle physiological status into bone-forming responses.

#### 3.5.2. Effects on OBs

Secretomes from healthy muscle stimulated preosteoblast viability, migration [31], proliferation, and differentiation of OBs and inhibited ferroptosis [40,41,43,48]. In some cases, muscle-derived secretomes also promoted OB-to-osteocyte transition [22,53]. Several studies demonstrated enhanced OB activity following exposure to EVs or CM derived from exercise or mechanically stimulated muscle [40,41,48]. Some studies further identified specific EV-associated mediators responsible for these effects. Myoblast-derived EVs delivering miR-27a-3p enhanced osteogenic differentiation through activation of β-catenin signaling [43], whereas irisin-enriched EVs promoted osteoblast proliferation and reduced ferroptosis via Caveolin-1-dependent AMPK (AMP-activated protein kinase) signaling [40]. Nevertheless, the evidence for irisin-enriched EVs is based on studies with lower methodological quality in both in vivo and in vitro assessments; therefore, these findings require confirmation in future research. Negative regulators included muscle-derived sclerostin, galectin-3, and muscle-specific Slo1 (big potassium channel) deficiency, which antagonized osteogenic differentiation [34,38,42]. Smooth muscle-derived EVs promoted OB-to-osteocyte maturation and concurrently increased TNF superfamily member 11 (TNFSF11, also known as RANKL) expression, thereby indirectly stimulating osteoclastogenesis [22].

#### 3.5.3. Effects on OCs

EVs from healthy or mechanically stimulated muscle generally suppressed OC formation, mitochondrial biogenesis, and bone resorption [20,31,44,45]. These effects were amplified by fluid flow shear stress [45]. One of the miRNAs responsible for inhibition of OC formation was miR-196a-5p [20,45]. In contrast, CM from estrogen-deficient or lamin A/C-deficient muscle promoted OC differentiation, activity, and osteoclastogenesis, while EVs from inflamed muscle blunted suppression of OC formation [5,33,46]. Huang et al. confirmed that EVs from healthy muscle inhibited OC formation, while those from atrophic muscle promoted it [35].

#### 3.5.4. In Vivo Confirmations

Multiple in vivo studies have demonstrated that CM or EVs from healthy or mechanically active muscle increased bone mass, improved bone repair and trabecular microarchitecture, and protected against bone loss in OVX, glucocorticoid-induced, or disuse-induced osteoporosis and diabetic models [16,23,31,32,35,36,37,39,41]. In contrast, in vivo exposure to secretome from atrophic [18] or functionally impaired muscle [33,42] inhibited osteogenic differentiation and promoted bone loss. These findings reflect the in vitro dichotomy and highlight the state-dependent directionality of muscle–bone crosstalk.

#### 3.5.5. Human-Relevant Evidence

Human data remain limited but consistent. Mechanically stimulated mouse myocytes promoted proliferation and osteogenic differentiation, while inhibiting OC formation in human bone marrow MSCs [16,39]. Human myotube-conditioned medium further modulated OB energy metabolism by increasing glucose uptake and reducing oleic acid uptake [47]. In two studies, Fernandes et al. investigated the effect of muscle-derived EVs on the transition of OBs to osteocytes [22,53]. Observational data also identified an association between EV cargo, such as BCL2-associated athanogene 6 (BAG6) and BMD [49]. No clinical trials of muscle secretome-based therapies were identified.

#### 3.5.6. Mechanistic Convergence

Across the included studies, several recurring pathways and cargos were identified, broadly categorized into anabolic and catabolic axes. Anabolic signaling included miR-92a-3p/PTEN/Akt [16], β-catenin [34,43], Prrx2-MIR22HG-Hippo/YAP [23] axis, and LDHA-associated glycolytic support [36]. In contrast, catabolic signaling involved Notch and Akt pathways (role of galectin-3) [38], TNF-α-altered EV cargo [46], estrogen-deficiency-associated myokines [5], and miR-222-3p [42]. Collectively, these mechanisms converge on a consistent directional pattern: healthy or mechanically stimulated muscle exhibits anabolic and antiresorptive effects, whereas atrophic or estrogen-deficient muscle exhibits catabolic or proresorptive effects. TNF-α stimulation of myoblasts also reduced the inhibitory effect of muscle-derived EVs on osteoclast formation and the stimulatory effect on osteoblast differentiation. Table 3 summarizes the experimental targets, type of intervention, mechanisms, and main outcomes.

Overall, across models, CM and EVs derived from healthy or mechanically stimulated muscle consistently promote osteogenic differentiation, enhance preosteoblast viability, proliferation, and migration, suppress OC formation and bone resorption in vitro, and increase bone mass or protect against bone loss in vivo. Conversely, secretome derived from dysfunctional muscle—including atrophic, estrogen-deficient, or lamin A/C-deficient muscle—consistently impairs osteogenic differentiation, promotes OC formation in vitro, and drives bone loss in vivo.

This dichotomy demonstrates a reproducible biological gradient: healthy/mechanically stimulated muscle promotes bone anabolic effects, while atrophic/estrogen-deficient muscle promotes bone catabolic effects. In addition, the protective effects of muscle-derived EVs on bone were attenuated under inflammatory conditions. This methodological consistency facilitates cross-model comparisons and underscores their translational relevance.

## 4. Discussion

Across 31 studies, the direction of muscle–bone biochemical crosstalk consistently mirrored the health state of skeletal muscle. Secretomes from healthy or mechanically stimulated muscle (CM and EVs) were osteoanabolic and antiresorptive, enhancing MSC proliferation, osteogenic differentiation, osteoblast function, and suppressing osteoclast formation [20,31,32,35,36,40,43,44], with multiple in vivo confirmations [23,31,32]. Conversely, secretomes from atrophic or estrogen-deficient muscle shifted signaling toward osteocatabolism, inhibiting osteogenic differentiation and promoting osteoclastogenesis, and secretomes from inflamed muscle blunted osteogenic effects [5,18,33,35]. This directional pattern was reproducible across models and preparations, indicating a robust biological gradient from muscle health to bone remodeling outcomes.

To date, no systematic review has specifically addressed the effects of the muscle-derived secretome on MSCs and bone cells. The existing literature has predominantly focused on studies investigating the influence of the MSC-derived secretome on other cell types or on secretomes from non-muscle sources [54,55,56]. A recent systematic review examined the effects of myokines on bone tissue metabolism, highlighting key factors such as irisin, myostatin, FGF-2, myonectin, IL-6, and IL-15, based on preclinical and clinical studies [24]. On the other hand, our systematic review, encompassing 18 in vivo and 31 in vitro studies, underscores the pivotal role of the muscle secretome in regulating bone MSC function and bone metabolism.

CM and EVs released from healthy muscles promote osteogenic differentiation, inhibit bone resorption, and enhance preosteoblast viability, proliferation, and migration in vitro. In vivo studies also demonstrate the beneficial effects of muscle-derived factors from healthy tissue on bone. Conversely, muscle-derived sclerostin has been shown to have detrimental effects on bone in both in vitro and in vivo conditions. In contrast, factors secreted by dysfunctional muscle—such as OVX muscle, skeletal muscle lacking estrogen receptor α (Erα) expression, or lamin A/C gene knockout myotubes—enhance OC differentiation and activity. These conditions were also associated with inhibited osteogenic differentiation in in vitro and in vivo models. MSCs, OBs, and OCs can internalize muscle-derived EVs, and their behavior reflects the physiological state of the donor muscle.

The mechanistic studies included in this review suggest that muscle-derived EVs are not merely biomarkers of muscle status but active regulators of MSC fate. Several EV cargos directly influence signaling pathways governing osteogenic commitment. For example, mechanically stimulated muscle-derived EVs enriched in miR-92a-3p suppress PTEN and activate Akt signaling, thereby enhancing MSC proliferation and osteogenic differentiation [16]. Likewise, exosomal Prrx2 promotes activation of the MIR22HG/miR-128/YAP1 axis, resulting in Hippo pathway activation and enhanced osteogenic differentiation [23]. Beyond gene regulation, EVs also transfer metabolic components, including glycolytic enzymes, which support the metabolic reprogramming required for osteoblast lineage commitment [36]. EVs derived from healthy muscle tissue promoted osteogenesis, whereas EVs derived from atrophic or dysfunctional muscle tissue contained inhibitory cargo, including miR-92a-1-5p [18] and miR-222-3p [42], which impaired osteogenic differentiation and shifted bone remodeling toward a catabolic state. Collectively, these findings indicate that EVs function as biologically active vectors that translate the physiological state of skeletal muscle into specific transcriptional and metabolic responses in bone-forming cells.

Importantly, the effects of muscle-derived factors on OBs are not universally positive. Johnson et al. demonstrated that myotube-CM from C2C12 myoblasts downregulated key OB differentiation markers, including ALP, Runx2 (RUNX family transcription factor 2), Osx (osterix), PTH (parathyroid hormone) receptor, OPG (osteoprotegerin), OC, and SOST (sclerostin), while upregulating RANKL, indicating a shift toward osteoclastogenic signaling [57]. Similarly, Magaro et al. [34] suggested that muscle-derived sclerostin contributes to the inhibition of OB maturation. In addition, galectin-3 was identified as a factor impairing early osteogenesis, and muscle-specific Slo1 deficiency was shown to inhibit osteogenic differentiation [38,42].

Although the study by Fulzele et al. [58] was not captured by our search strategy, it provides relevant complementary evidence. The authors demonstrated an age-associated increase in miR-34a in EVs released from C2C12 myoblasts and primary human myotubes. Notably, miR-34a-enriched EVs decreased the viability of bone marrow MSCs and induced their senescence. Furthermore, EVs from miR-34a-overexpressing C2C12 cells reduced Sirt1 expression ex vivo.

Human evidence remains limited but directionally consistent. Studies using human primary myotubes and human bone marrow MSCs show that mechanically stimulated muscle CM promotes MSC proliferation and osteogenic differentiation, while also modulating OB energy metabolism. Human smooth muscle biopsy-derived EVs influence the OB-to-osteocyte transition. Correlative studies link circulating muscle-related EV markers, such as BAG6, to BMD. However, no clinical trials have directly tested muscle secretome-based therapies, underscoring the preclinical status of this field.

**Figure 2:**
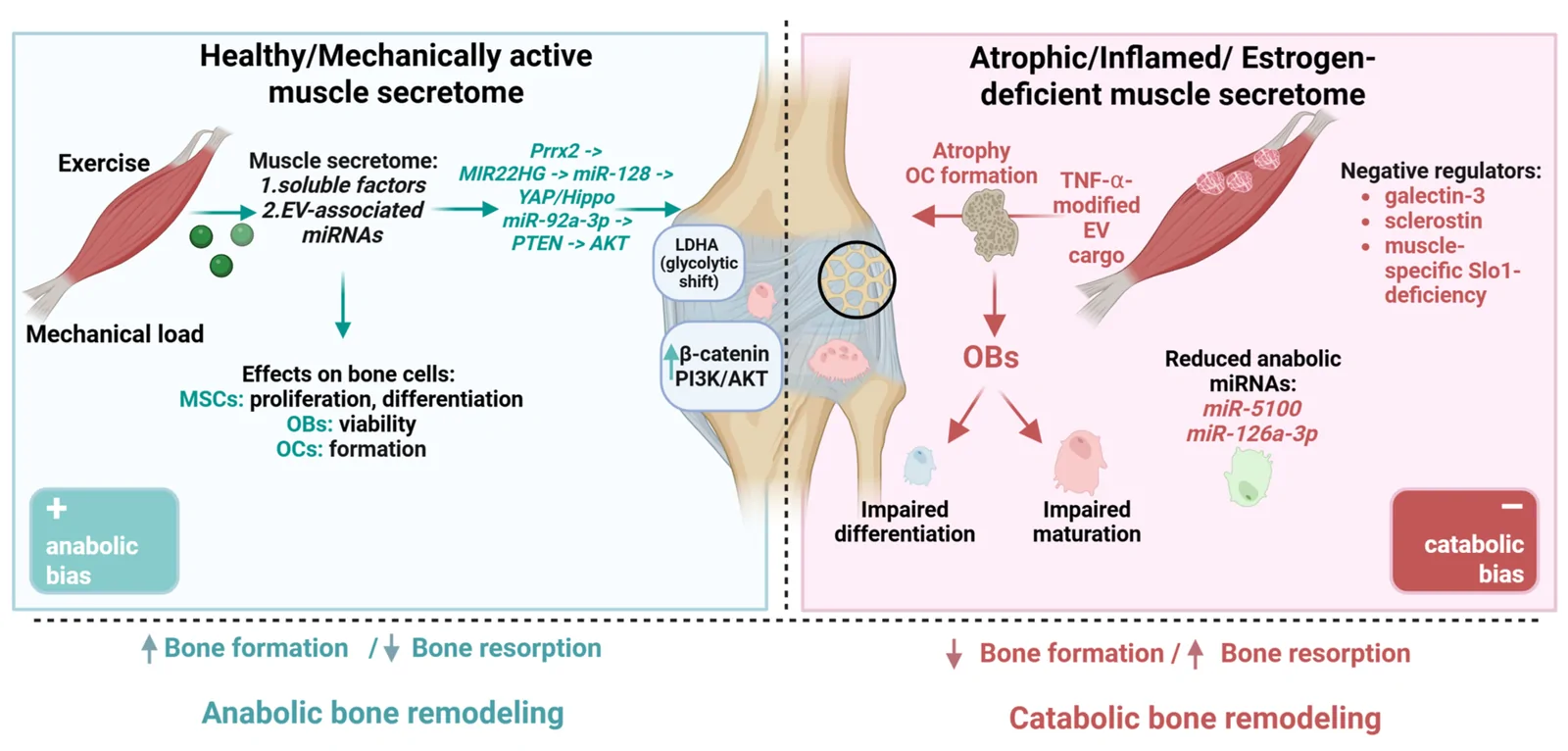
Schematic representation of the main findings of this study. The predominant reported effects of muscle-derived mediators on bone are summarized. Healthy or mechanically active muscle delivers osteogenic or bone-anabolic stimuli, whereas atrophic or estrogen-deficient muscle promotes osteoresorptive or bone-catabolic signaling, and inflamed muscle blunts the osteogenic effects. Some factors, including galectin-3, may exert context-dependent effects and, therefore, their placement reflects the net biological effect reported in the included studies. An upward-pointing arrow indicates an increase and a downward-pointing arrow indicates a decrease. Created in BioRender. (2026) https://BioRender.com/glqajsa.

Beyond differentiation signals, the muscle secretome influences bone cell metabolism (Figure 2). Myotube-derived CM alters OB energy usage by increasing glucose uptake and reducing fatty acid utilization. Such metabolic reprogramming may become particularly relevant under conditions of aging, systemic inflammation, or metabolic disease, where energetic demands shape bone cell survival and function. These findings highlight a bidirectional interplay between muscle-derived metabolic cues and bone-cell energetics.

### 4.1. Translational Implications

Given that osteosarcopenia likely reflects impaired mechanobiochemical crosstalk, our synthesis supports two complementary translational routes: (a) exercise-based or exerkine strategies to recondition the muscle secretome toward an anabolic/antiresorptive profile—potentially trackable by EV cargo biomarkers—for prevention or adjunct therapy in osteoporosis or osteosarcopenia; (b) EV-based or EV-mimetic therapeutics engineered to deliver osteoanabolic cargo (e.g., miR-92a-3p, Prrx2 axis, LDHA), informed by state-dependent mechanistic mapping. Early preclinical work shows bone-healing and anti-loss signals from healthy or mechanically stimulated muscle EVs, motivating standardized, safety-first translation pathways.

Based on our experience, most studies neither cite MISEV publications nor adhere to the reporting requirements, despite the guidelines being developed through broad consensus with the community. While there is still considerable room for improvement, increased attention to precise reporting and citation in accordance with MISEV guidelines would enhance rigor, reproducibility, and transparency in the field [59].

However, translation into clinical therapies requires substantial methodological refinement, including standardized EV isolation, MISEV-aligned characterization, cargo profiling, purity metrics, dose normalization, and rigorous safety assessment. Preclinical studies demonstrate that EVs from healthy or mechanically stimulated muscle enhance osteogenesis and bone healing, supporting the feasibility of EV-based therapies for osteoporosis, fracture non-unions, and age-related bone loss.

### 4.2. Contribution of the Systematic Review

A major contribution of this review is its emphasis on whole secretome effects (CM and EVs), which more accurately reflect physiological paracrine communication than isolated myokines. Many individual myokines underpredict the robust osteogenic signals observed with whole secretome preparations, likely because bone cells are exposed to complex combinations of soluble factors and packaged EV cargo. Aligning nomenclature with MISEV2023 standards helps avoid mechanistic overspecification, particularly regarding “exosome-specific” claims.

The reviewed studies highlight the dual role of the muscle-derived secretome in bone remodeling—stimulating osteogenesis and suppressing resorption under healthy conditions but reversing these effects in pathological states. Identification of specific miRNAs and signaling pathways (such as miR-92a-3p/PTEN/Akt, β-catenin, Notch, and PI3K/Akt) provides mechanistic insight and potential therapeutic targets. Given the age-related decline in muscle function and secretory profile, interventions to restore or mimic a healthy muscle secretome—such as exercise-induced myokine modulation or engineered EVs—may offer novel strategies for treating osteoporosis and osteosarcopenia.

Nevertheless, some secreted factors may exert context-dependent effects. For example, galectin-3 has been reported to be secreted during normal myogenic differentiation and mechanical stimulation of C2C12 myoblasts, while also impairing osteogenic differentiation in target bone cells. The classification presented in Figure 2 reflects the predominant bone-related effects of mediators from the muscle-derived secretome.

### 4.3. Limitations of Current Evidence and Future Directions

Strengths of the included literature are reflected in the convergent directionality across multiple models, cell types, and secretome formats (CM and EVs), with in vivo confirmation in diverse bone-loss paradigms (OVX, glucocorticoids, disuse, diabetes). This reproducibility increases confidence in the underlying biological hypothesis. However, in vivo studies frequently lacked adequate justification for selection, performance, and detection biases. Attrition bias was also insufficiently reported in most cases, whereas reporting bias and other sources of bias were described more consistently. In vitro studies demonstrated higher methodological quality, though blinding and management of incomplete outcome data were often unclear. Limitations are substantive: (i) heterogeneity in muscle sources (cell lines vs. tissue), preparation types (CM vs. EVs; frequent use of “exosomes” by authors), characterization depth, dosing/normalization, interventions, comparators, outcome measures, and reporting formats; (ii) in vivo RoB concerns (allocation concealment, housing/randomization, blinding, incomplete outcome data) and in vitro gaps (sample size justification, blinding, incomplete outcome data handling); (iii) irisin-enriched EV evidence is methodologically limited; (iv) the miR-92a family requires careful differentiation (robust data for miR-92a-3p, preliminary for miR-92a-1-5p); and (v) few human studies and no clinical trials to date. Accordingly, the qualitative classification used in this review reflects the predominant biological effects reported within individual studies and should not be interpreted as a quantitative measure of effect size or consistency across studies. These constraints caution against overgeneralization and emphasize the necessity of standardized, mechanistic, and translationally guided study designs.

A further limitation of the current literature is that relatively few studies have experimentally distinguished EV-associated effects from those mediated by soluble factors. Consequently, although evidence supports a role for both components in muscle-to-bone communication, their relative importance remains incompletely defined. Future studies using EV-depleted CM or cargo-specific manipulations will be required to determine whether the observed biological effects are primarily driven by soluble factors, EVs, or synergistic interactions between the two components.

Future research priorities should include: (a) MISEV2023-aligned reporting and standardization of EV isolation, purity assessment, cargo profiling, and dose normalization; (b) mechanistic resolution through gain-/loss-of-function experiments probing the necessity and sufficiency of key cargo (e.g., miR-92a-3p, Prrx2/MIR22HG, LDHA); (c) human prospective studies linking exercise-induced changes in secretome/EV cargo to bone outcomes; (d) early-phase clinical development of EV-mimetic therapeutics with safety, pharmacokinetic/pharmacodynamic, and biomarker-guided endpoints, and (e) integration with endocrine and inflammatory axes, especially estrogen deficiency and TNF-α-mediated muscle inflammation, to inform personalized strategies for osteoporosis and osteosarcopenia.

## 5. Conclusions

Healthy or mechanically stimulated muscle produces a secretome that promotes osteogenic differentiation and suppresses bone resorption, whereas atrophic or estrogen-deficient muscle releases factors that reverse this balance. These directionally consistent observations across cell types and animal models support the development of exercise-based interventions and EV-based therapeutics for osteoporosis and osteosarcopenia. However, clinical translation requires rigorous MISEV-aligned standardization, mechanistic validation, and robust human studies.

## Figures and Tables

**Table 1:** Risk of bias for in vivo studies using SYRCLE’s RoB tool.

	Selection Bias: 1–3			Performance Bias: 4–5		Detection Bias: 6–7		Attrition Bias	Reporting Bias	Other
Study	Item 1	Item 2	Item 3	Item 4	Item 5	Item 6	Item 7	Item 8	Item 9	Item 10
Lee et al., 2019 [31]	Unclear	Unclear	Unclear	Unclear	Unclear	Unclear	Yes	No	No	No
Saiz et al., 2019 [32]	Unclear	Unclear	Unclear	Unclear	Unclear	Unclear	Yes	No	Yes	No
Xiong et al., 2020 [33]	Unclear	Unclear	Unclear	Unclear	Unclear	Unclear	Yes	No	No	Yes
Magarò et al., 2021 [34]	Yes	Unclear	Unclear	Unclear	Unclear	Unclear	Unclear	No	No	Yes
Andrew Norton et al., 2022 [5]	Unclear	Unclear	Unclear	Unclear	Unclear	Unclear	Unclear	Yes	Yes	Yes
Huang et al., 2023 [35]	Yes	Yes	No	Unclear	No	Unclear	Yes	No	Yes	Yes
Lara-Castillo et al., 2023 [21]	Unclear	Unclear	Unclear	Unclear	Unclear	Unclear	Yes	No	Yes	Yes
Li et al., 2023 [23]	Yes	Unclear	Unclear	Unclear	Unclear	Unclear	Unclear	Yes	Yes	No
Ma et al., 2023 [36]	Unclear	Yes	Unclear	Unclear	Unclear	Unclear	Unclear	Yes	Yes	Yes
Takafuji et al., 2023 [37]	Unclear	Yes	Unclear	Unclear	Unclear	Unclear	Unclear	No	No	Yes
Xu et al., 2023 [16]	Yes	Yes	Yes	Yes	Unclear	Unclear	Yes	No	Yes	Yes
Amore et al., 2024 [38]	Unclear	Unclear	Unclear	Unclear	Unclear	Unclear	Unclear	No	Yes	Yes
W. Chen et al., 2024 [19]	Yes	Unclear	Unclear	Unclear	Unclear	Unclear	Unclear	No	Yes	Yes
M. Chen et al., 2024 [39]	Unclear	Unclear	Unclear	Unclear	Unclear	Unclear	Unclear	No	Yes	Yes
Tao et al., 2024 [40]	Unclear	Unclear	Unclear	Unclear	Unclear	Unclear	Unclear	Yes	No	No
Xing et al., 2024 [41]	Yes	Unclear	Unclear	Unclear	Unclear	Yes	Unclear	No	Yes	Yes
B. Wang et al., 2025 [18]	Unclear	Unclear	Unclear	Unclear	Unclear	Unclear	Unclear	No	Yes	Yes
Y. Wang et al., 2025 [42]	Unclear	Unclear	Unclear	Unclear	Unclear	Unclear	Unclear	No	Yes	Yes

Green = low risk of bias; yellow = unclear risk of bias; red = high risk of bias.

**Table 2:** Risk of bias for in vitro studies using modified SYRCLE’s RoB tool.

	Selection Bias: 1–2		Performance Bias: 3–4		Detection Bias: 5–7			Attrition Bias	Reporting Bias	Other
Study	Item 1	Item 2	Item 3	Item 4	Item 5	Item 6	Item 7	Item 8	Item 9	Item 10
Xu et al., 2018 [43]	No	Yes	No	Yes	Yes	Yes	Unclear	Unclear	Yes	No
Lee et al., 2019 [31]	Yes	Yes	Yes	Yes	Yes	Yes	Unclear	Unclear	No	No
Saiz et al., 2019 [32]	No	Yes	Yes	Yes	Yes	Yes	Unclear	Unclear	Yes	No
Takafuji et al., 2020 [44]	No	Yes	Yes	Yes	Yes	Yes	Unclear	Unclear	Yes	No
Xiong et al., 2020 [33]	No	Yes	Yes	Yes	Yes	No	Unclear	Unclear	No	Yes
Magarò et al., 2021 [34]	No	Yes	Yes	Yes	Yes	Yes	Unclear	Unclear	No	Yes
Takafuji et al., 2021 [20]	No	Yes	Yes	Yes	Yes	Yes	Unclear	Unclear	Yes	Yes
Takafuji et al., 2021 [45]	No	Yes	Yes	Yes	Yes	Yes	Unclear	Unclear	Yes	Yes
Andrew Norton et al., 2022 [5]	Yes	Yes	Yes	Yes	Yes	Yes	Unclear	Yes	Yes	Yes
Takada et al., 2022 [46]	No	Yes	Yes	Yes	Yes	Yes	Unclear	Unclear	Yes	Yes
Huang et al., 2023 [35]	No	Yes	Yes	Yes	Yes	Yes	Unclear	Unclear	Yes	Yes
Lara-Castillo et al., 2023 [21]	No	Yes	Yes	Yes	Yes	Yes	Unclear	Unclear	Yes	Yes
Li et al., 2023 [23]	No	Yes	Yes	Yes	Yes	Yes	Unclear	Unclear	Yes	No
Lunde et al., 2023 [47]	Yes	Yes	Yes	Yes	Yes	Yes	Unclear	Unclear	Yes	Yes
Ma et al., 2023 [36]	No	Yes	Yes	Yes	Yes	Yes	Unclear	Yes	Yes	Yes
Suresh Kumar et al., 2023 [48]	No	Yes	Yes	Yes	Yes	Yes	Unclear	Unclear	Yes	Yes
Takafuji et al., 2023 [37]	No	Yes	Yes	Yes	Yes	Yes	Unclear	Unclear	No	Yes
Xu et al., 2023 [16]	No	Yes	Yes	Yes	Yes	Yes	Unclear	Unclear	Yes	Yes
Amore et al., 2024 [38]	No	Yes	Yes	Yes	Yes	Yes	Unclear	Unclear	Yes	Yes
W. Chen et al., 2024 [19]	No	Yes	Yes	Yes	Yes	Yes	Unclear	Unclear	Yes	Yes
M. Chen et al., 2024 [39]	No	Yes	Yes	Yes	Yes	Yes	Unclear	Unclear	Yes	Yes
Fernandes et al., 2024 [22]	No	Yes	Yes	Yes	Yes	Yes	Unclear	Unclear	Yes	Yes
Tao et al., 2024 [40]	No	Yes	Yes	Yes	Yes	No	Unclear	Unclear	No	No
Xing et al., 2024 [41]	No	Yes	Yes	Yes	Yes	No	Unclear	Unclear	Yes	Yes
Yin et al., 2024 [49]	No	Yes	Yes	No	Yes	Yes	Unclear	Unclear	Yes	No
Chen et al., 2025 [50]	No	Yes	Yes	Yes	Yes	Yes	Unclear	Unclear	Yes	Yes
Tu-Shen Chen et al., 2025 [51]	No	Yes	Yes	Yes	Yes	Yes	Unclear	Unclear	Yes	Yes
Hoang et al., 2026 [52]	No	Yes	Yes	Yes	Yes	Yes	Unclear	Unclear	Yes	Yes
B. Wang et al., 2025 [18]	No	Yes	Yes	Yes	Yes	Yes	Unclear	Unclear	Yes	Yes
Y. Wang et al., 2025 [42]	No	Yes	Yes	Yes	Yes	Yes	Unclear	Unclear	Yes	Yes
Fernandes et al., 2026 [53]	Yes	Yes	Yes	Yes	Yes	Yes	Unclear	Unclear	Yes	Yes

Green = low risk of bias; yellow = unclear risk of bias; red = high risk of bias.

**Table 3:** Summary of the included studies.

Author/Year	Target Animals/Cells	Type of Intervention ^a^	Mechanism/Signaling Pathway/Key miRNA	Main Outcome ^b^
M. Chen et al., 2024 [39]	C57BL/6J mice; hBMSCs	EVs	miR-873-3p	↑ osteogenic gene expression and osteogenic differentiation, ↓ osteoclast formation in vitro; ↑ bone mass in vivo
Tu-Shen Chen et al., 2025 [51]	Mouse BMSCs line D1	CM	/	↑ osteogenic differentiation
Hoang et al., 2026 [52]	iMSCs	CM	/	↑ osteogenic differentiation
Lee et al., 2019 [31]	C57BL/6 mice; BMM, MC3T3-E1, primary mouse OBs	CM	/	↓ bone resorption, ↑ preosteoblast activity and migration in vitro; ↑ bone mass in vivo
Li et al., 2023 [23]	C57BL/6J mice; mouse BMSCs	EVs ^c^	Prrx2 → MIR22HG → Hippo/YAP pathway	↑ YAP signaling and osteogenic differentiation in vitro; ↓ bone loss in vivo
Ma et al., 2023 [36]	C57BL/6J mice; mouse BMSCs	EVs	LDHA-containing EVs, glycolysis-associated signaling	Reprograms MSC metabolism toward glycolysis, providing energy for ↑ osteogenic differentiation in vitro; ↓ bone loss in vivo
Saiz et al., 2019 [32]	SD rats; rBMSCs	CM	/	↑ osteogenic differentiation in vitro; ↑ bone formation in vivo
Takafuji et al., 2021 [20]	RAW 264.7	EVs	Mitochondrial energy metabolism, miR-196a-5p	↓ OC formation and mitochondrial energy metabolism, ↑ osteogenic differentiation
Takafuji et al., 2020 [44]	Mouse bone marrow cells, RAW 264.7	EVs	Mitochondrial energy metabolism	↓ OC formation
Takafuji et al., 2021 [45]	RAW 264.7	Myo-EV, FFSS-Myo-EV	miR-155-5p, miR-196a-5p	↓ OC formation
Takafuji et al., 2023 [37]	C57BL/6J mice; ST-2	EVs	/	↑ osteogenic differentiation in vitro; ↑ bone repair (analysis of bone defect area, BV/TV, mRNA levels of osteogenic markers) in vivo
Tao et al., 2024 [40]	C57BL/6J mice; MC3T3-E1	CM and EVs	AMPK pathway	↑ proliferation, ↓ ferroptosis and ↑ differentiation of OBs in vitro
Xing et al., 2024 [41]	SD rats; MC3T3-E1	EVs ^c^	/	↑ osteogenic differentiation in vitro and in vivo (trabeculae analysis (BV/TV, Tb.N, Tb.Th, Tb.Sp))
Xu et al., 2023 [16]	C57BL/6J mice; hBMSCs	EVs ^c^	PTEN/Akt pathway, miR-92a-3p	Inhibits PTEN, activates Akt signaling, ↑ MSC proliferation and osteogenic differentiation in vitro; ↓ bone loss in vivo
Xu et al., 2018 [43]	MC3T3-E1	EVs ^c^	β-catenin pathway, miR-27a-3p	↑ osteogenic differentiation
W. Chen et al., 2024 [19]	SAMP6 mice; mouse BMSCs	EVs ^c^	miR-5100, miR-126a-3p, miR-450b-5p, miR-669a-5p	↑ osteogenic differentiation in vitro
				↑ miRNAs and osteogenesis-related TFs (OCN, Osterix) expression in vivo
Huang et al., 2023 [35]	C57BL/6J mice; mouse BMSCs, BMM, mouse OBs	Healthy vs. atrophic muscle EVs	/	EVs from normal skeletal muscle: ↑ osteogenic differentiation and ↓ OC formation in vitro; ↑ bone formation (measurement of bone mass) and ↓ bone resorption (trabeculae analysis) in vivo
				EVs from atrophic skeletal muscle: ↓ osteogenic differentiation and ↑ OC formation in vitro
Amore et al., 2024 [38]	MC3T3	CM	Notch and Akt pathway	↓ osteogenic differentiation
Magarò et al., 2021 [34]	C57BL/6 mice; 2T3	CM	Wnt/β-catenin pathway	↓ OB differentiation in vitro; inhibitory role on bones in vivo (trabecular analysis (loss of trabecular bone), change in metaphyseal trabeculae)
Andrew Norton et al., 2022 [5]	Primary mouse OCs	CM	Estrogen signaling in muscle	↑ OC differentiation and activity in vitro
Takada et al., 2022 [46]	Mouse BM cells, RAW 264.7, ST-2	EVs	miR-196-5p	Blunted ↓ OC formation and promotion of OB differentiation
B. Wang et al., 2025 [18]	SD rats; C3H10 T1/2	EVs ^c^	miR-92a-1-5p	↓ osteogenic differentiation in vitro and in vivo
Y. Wang et al., 2025 [42]	C57BL/6 mice; MC3T3-E1	EVs ^c^	miR-222-3p/STAT3 pathway	↓ osteogenic differentiation in vitro; ↓ bone mass in vivo
Xiong et al., 2020 [33]	Lmna^HSA^-cko mice; primary mouse BMMs (co-culture with OBs)	CM	/	↑ osteoclastogenesis in vitro; ↑ bone loss (trabeculae and cortical analysis) and serum levels of markers OCN and PYD in vivo
Chen et al., 2025 [50]	MC3T3-E1	EVs ^c^	/	↔ expression of genes PDGFRB, COL12A1, TBX2, C3
Fernandes et al., 2024 [22]	Primary human OBs	CM	β-catenin pathway, miR-23a	↑ transition of OBs to osteocytes
Fernandes et al., 2026 [53]	Human MSCs, primary human OBs	CM and EVs	SOST, DMP1, miR-23a	↑ OB-to-osteocyte transition
Lara-Castillo et al., 2023 [21]	TOPGAL mice; MLO-Y4	CM	PI3K/Akt and β-catenin pathways	Response of osteocytes to mechanical loading in vitro, ex vivo and in vivo
Lunde et al., 2023 [47]	hBMSCs (differentiated to OBs)	CM	/	↑ glucose uptake and ↓ oleic acid uptake
Suresh Kumar et al., 2023 [48]	MC3T3	CM	/	↑ osteogenic markers ALP, Col1a1 and Spp1
Yin et al., 2024 [49]	Human BM cells, PBMCs (Th17/macrophage differentiation assays)	EVs ^c^	NF-κB pathway	Clinical: negative correlation between serum exosomal BAG6 and BMD

Abbreviations: ↔: change; 2T3: osteoblastic cell line; ALP: alkaline phosphatase; AMPK: AMP-activated protein kinase; BAG6: BCL2-associated athanogene 6; BM: bone marrow; BMD: bone mineral density; BMM: bone marrow macrophages; BMSCs: bone marrow MSCs; BV/TV: bone volume to total volume ratio; C3: complement component 3; C3H10 T1/2: mouse embryonic cell line, exhibiting fibroblast morphology; CM: conditioned medium; Col1a1: collagen type I alpha 1 chain; COL12A1: collagen type XII alpha 1 chain; DMP1: dentin matrix protein 1; EVs: extracellular vesicles; FFSS: fluid flow shear stress; FFSS-Myo-EV: EVs secreted from C2C12 cells exposed with FFSS; hBMSCs: human bone marrow MSCs; HSA: human alpha-skeletal actin; iMSCs: induced pluripotent stem cell-derived MSCs; LMNA: lamin A/C gene; Lmna^HSA^-cko: skeletal muscle-specific Lmna conditional knockout mice; MC3T3-E1: murine preosteoblasts; MLO-Y4: murine osteocyte-like cells; MSCs: mesenchymal stem/stromal cells; Myo-EV: EVs from mouse muscle C2C12 cells; NF-κB: nuclear factor κB; OB(s): osteoblast(s); OC(s): osteoclast(s); OCN: osteocalcin; PBMCs: human peripheral blood monocytes; PDGFRB: platelet-derived growth factor receptor beta; PYD: pyridinoline; RAW 264.7: mouse monocytic cells; rBMSCs: rat bone marrow stromal cells; SAMP6: senescence-accelerated mouse prone 6 strain of mice; SD: Sprague-Dawley; Spp1: osteopontin; SOST: sclerostin; STAT3: signal transducer and activator of transcription 3; ST-2: mouse mesenchymal stromal cells; Tb.N: trabecular number; Tb.Sp: trabecular spacing; Tb.Th: trabecular thickness; TBX2: T-box transcription factor 2; TF: transcription factor; Th17: T helper 17. ^a^ CM vs. EVs vs. exosomes. ^b^ Green = net anabolic/antiresorptive; red = net catabolic/proresorptive; without color = other effects; ↑ indicates an increase, promotion, enhancement, improvement, or stimulation, whereas ↓ indicates a decrease, inhibition, reduction, suppression, attenuation, or amelioration, as applicable. ^c^ The authors used “exosomes,” relabeled here as EVs, because no biogenesis-specific evidence was provided.

## Data Availability

No new data were created or analyzed in this study. Data sharing is not applicable to this article.

## Supplementary Materials

The following supporting information can be downloaded at https://www.mdpi.com/article/10.3390/ijms27209102/s1.

## Abbreviations

The following abbreviations are used in this manuscript:

Akt	Protein kinase B
ALP	Alkaline phosphatase
AMPK	AMP-activated protein kinase
ARS	Alizarin red S
ASySD	Automated Systematic Search Deduplicator
BMD	Bone mineral density
CM	Conditioned medium
EVs	Extracellular vesicles
FGF-2	Fibroblast growth factor 2
IL	Interleukin
LDHA	Lactate dehydrogenase A
micro-CT	Micro-computed tomography
MIR22HG	MIR22 host gene
miRNA	MicroRNA
MISEV2023	Minimal information for studies of extracellular vesicles 2023
MSCs	Mesenchymal stem/stromal cells
OBs	Osteoblasts
OCs	Osteoclasts
OPG	Osteoprotegerin
Osx	Osterix
OVX	Ovariectomy
PI3K	Phosphatidylinositol 3-kinase
PICO	Population, Intervention, Comparison, Outcome
PRISMA	Preferred Reporting Items for Systematic Reviews and Meta-Analyses
Prrx2	Paired-related homeobox 2
PTEN	Phosphatase and tensin homolog
PTH	Parathyroid hormone
RANKL	Receptor activator of nuclear kappa-B ligand
RoB	Risk of bias
Runx2	RUNX family transcription factor 2
Slo1	Big potassium channel
SOST	Sclerostin
SYRCLE	Systematic Review Center for Laboratory Animal Experimentation
TNF-α	Tumor necrosis factor-α
TNFSF11	TNF superfamily member 11
TRAP	Tartrate-resistant acid phosphatase
YAP	Yes-associated protein
