# Supplementary material for: Directional Muscle-to-Bone Crosstalk via the Muscle Secretome: A Systematic Review
Source: Int J Mol Sci. 2026 Oct 14;27(20):9102. doi: 10.3390/ijms27209102 (PMC13704898; doi:10.3390/ijms27209102)
Supplement: Supplementary file 1 [file ijms-27-09102-s001.zip › Supplementary Material_Tables S1, S2, S3, S4, S5, S6 and S7.pdf]

Supplementary Material

Table S1. PRISMA checklist.

Section and Topic	Item #	Checklist item	Location where item is reported
**TITLE**			
Title	1	Identify the report as a systematic review.	Page 1
**ABSTRACT**			
Abstract	2	See the PRISMA 2020 for Abstracts checklist.	Page 1
**INTRODUCTION**			
Rationale	3	Describe the rationale for the review in the context of existing knowledge.	Pages 2–3
Objectives	4	Provide an explicit statement of the objective(s) or question(s) the review addresses.	Pages 2–3
**METHODS**			
Eligibility criteria	5	Specify the inclusion and exclusion criteria for the review and how studies were grouped for the syntheses.	Page 4 Section 2.2
Information sources	6	Specify all databases, registers, websites, organisations, reference lists and other sources searched or consulted to identify studies. Specify the date when each source was last searched or consulted.	Pages 3–4 Section 2.1
Search strategy	7	Present the full search strategies for all databases, registers and websites, including any filters and limits used.	Pages 3–4 Section 2.1 Tables S2 and S3
Selection process	8	Specify the methods used to decide whether a study met the inclusion criteria of the review, including how many reviewers screened each record and each report retrieved, whether they worked independently, and if applicable, details of automation tools used in the process.	Page 4 Sections 2.2 and 2.3
Data collection process	9	Specify the methods used to collect data from reports, including how many reviewers collected data from each report, whether they worked independently, any processes for obtaining or confirming data from study investigators, and if applicable, details of automation tools used in the process.	Page 4 Section 2.3
Data items	10a	List and define all outcomes for which data were sought. Specify whether all results that were compatible with each outcome domain in each study were sought (e.g. for all measures, time points, analyses), and if not, the methods used to decide which results to collect.	Pages 4–5 Sections 2.4 and 2.5
	10b	List and define all other variables for which data were sought (e.g. participant and intervention characteristics, funding sources). Describe any assumptions made about any missing or unclear information.	Page 4 Section 2.4
Study risk of bias	11	Specify the methods used to assess risk of bias in the included studies, including details of the tool(s) used, how many reviewers assessed each	Page 5

Section and Topic	Item #	Checklist item	Location where item is reported
assessment		study and whether they worked independently, and if applicable, details of automation tools used in the process.	Section 2.6
Effect measures	12	Specify for each outcome the effect measure(s) (e.g. risk ratio, mean difference) used in the synthesis or presentation of results.	NA
Synthesis methods	13a	Describe the processes used to decide which studies were eligible for each synthesis (e.g. tabulating the study intervention characteristics and comparing against the planned groups for each synthesis (item #5)).	Page 4<br>Sections 2.2, 2.3, 2.4
	13b	Describe any methods required to prepare the data for presentation or synthesis, such as handling of missing summary statistics, or data conversions.	NA
	13c	Describe any methods used to tabulate or visually display results of individual studies and syntheses.	Pages 4–5<br>Sections 2.4 and 2.5
	13d	Describe any methods used to synthesize results and provide a rationale for the choice(s). If meta-analysis was performed, describe the model(s), method(s) to identify the presence and extent of statistical heterogeneity, and software package(s) used.	Page 5<br>Section 2.8
	13e	Describe any methods used to explore possible causes of heterogeneity among study results (e.g. subgroup analysis, meta-regression).	NA
	13f	Describe any sensitivity analyses conducted to assess robustness of the synthesized results.	NA
Reporting bias assessment	14	Describe any methods used to assess risk of bias due to missing results in a synthesis (arising from reporting biases).	Page 5<br>Section 2.6
Certainty assessment	15	Describe any methods used to assess certainty (or confidence) in the body of evidence for an outcome.	NA
**RESULTS**			
Study selection	16a	Describe the results of the search and selection process, from the number of records identified in the search to the number of studies included in the review, ideally using a flow diagram.	Pages 5–6<br>Section 3.1<br>Figure 2
	16b	Cite studies that might appear to meet the inclusion criteria, but which were excluded, and explain why they were excluded.	Pages 5–6<br>Section 3.1<br>Figure 2, Table S7
Study characteristics	17	Cite each included study and present its characteristics.	Pages 6, 11–14<br>Section 3.2<br>Tables 3 and S6
Risk of bias in	18	Present assessments of risk of bias for each included study.	Pages 7–9

Section and Topic	Item #	Checklist item	Location where item is reported
studies			Section 3.3<br>Tables 1 and 2
Results of individual studies	19	For all outcomes, present, for each study: (a) summary statistics for each group (where appropriate) and (b) an effect estimate and its precision (e.g. confidence/credible interval), ideally using structured tables or plots.	NA
Results of syntheses	20a	For each synthesis, briefly summarise the characteristics and risk of bias among contributing studies.	Pages 6–14<br>Sections 3.2, 3.3 and 3.5<br>Tables 1–3
	20b	Present results of all statistical syntheses conducted. If meta-analysis was done, present for each the summary estimate and its precision (e.g. confidence/credible interval) and measures of statistical heterogeneity. If comparing groups, describe the direction of the effect.	NA
	20c	Present results of all investigations of possible causes of heterogeneity among study results.	NA
	20d	Present results of all sensitivity analyses conducted to assess the robustness of the synthesized results.	NA
Reporting biases	21	Present assessments of risk of bias due to missing results (arising from reporting biases) for each synthesis assessed.	Pages 7–9<br>Section 3.3<br>Tables 1 and 2
Certainty of evidence	22	Present assessments of certainty (or confidence) in the body of evidence for each outcome assessed.	NA
**DISCUSSION**			
Discussion	23a	Provide a general interpretation of the results in the context of other evidence.	Pages 14–17<br>Section 4
	23b	Discuss any limitations of the evidence included in the review.	Pages 16–17<br>Section 4
	23c	Discuss any limitations of the review processes used.	Pages 16–17<br>Section 4
	23d	Discuss implications of the results for practice, policy, and future research.	Page 17<br>Section 4
**OTHER INFORMATION**			

Section and Topic	Item #	Checklist item	Location where item is reported
Registration and protocol	24a	Provide registration information for the review, including register name and registration number, or state that the review was not registered.	Page 3 Section 2
	24b	Indicate where the review protocol can be accessed, or state that a protocol was not prepared.	Page 3 Section 2
	24c	Describe and explain any amendments to information provided at registration or in the protocol.	NA
Support	25	Describe sources of financial or non-financial support for the review, and the role of the funders or sponsors in the review.	Page 18 Funding section
Competing interests	26	Declare any competing interests of review authors.	Page 18 Conflict of Interest section
Availability of data, code and other materials	27	Report which of the following are publicly available and where they can be found: template data collection forms; data extracted from included studies; data used for all analyses; analytic code; any other materials used in the review.	Page 18 Data Availability Statement section

Abbreviation: NA: not applicable.

From: Page MJ, McKenzie JE, Bossuyt PM, Boutron I, Hoffmann TC, Mulrow CD, et al. The PRISMA 2020 statement: an updated guideline for reporting systematic reviews. BMJ 2021;372:n71. doi: 10.1136/bmj.n71. This work is licensed under CC BY 4.0. To view a copy of this license, visit https://creativecommons.org/licenses/by/4.0/

Table S2. PICO characteristics in this systematic review.

PARAMETER	INCLUSION
Population	Human/animal/cell models
Intervention	Muscle secretome/CM/EVs/exosomes
Comparator	Treated vs. untreated MSCs or bone cells
Outcomes	Osteogenesis/osteoclastogenesis and related molecular/functional readouts

Table S3. Search strategy in specific databases based on full Boolean strategies.

**Database**	**Query**
Pubmed	((conditioned medium OR secretome OR secretory profile OR secretory factors OR myokine OR myokine* OR extracellular vesicles OR exosomes OR secreted factors) AND muscle)<br>AND (((MSC OR mesenchymal stem cells OR mesenchymal stromal cells OR bMSC OR BMSC) AND bone) OR (bone cells OR osteoblasts OR osteoclasts OR osteocytes))<br>AND (cytokines OR interleukins OR growth factors OR immunomodulation OR paracrine signaling OR signaling pathway* OR phenotype OR metabolism OR cell proliferation OR cell differentiation OR mineralization OR transcriptome* OR transcription OR proteome OR metabolome OR gene expression OR multiomics OR integrative)<br>AND (bone* OR osteoporosis OR sarcopenia OR osteosarcopenia)
Web of Science	((conditioned medium OR secretome OR secretory profile OR secretory factors OR myokine OR myokine* OR extracellular vesicles OR exosomes OR secreted factors) AND muscle)<br>AND (((MSC OR mesenchymal stem cells OR mesenchymal stromal cells OR bMSC OR BMSC) AND bone) OR (bone cells OR osteoblasts OR osteoclasts OR osteocytes))<br>AND (cytokines OR interleukins OR growth factors OR immunomodulation OR paracrine signaling OR signaling pathway* OR phenotype OR metabolism OR cell proliferation OR cell differentiation OR mineralization OR transcriptome* OR transcription OR proteome OR metabolome OR gene expression OR multiomics OR integrative)<br>AND (bone* OR osteoporosis OR sarcopenia OR osteosarcopenia)

Table S4. SYRCLE's tool for assessing risk of bias in *in vivo* research studies.

**Item**	**Type of bias**	**Domain**	**Description of domain**	**Review authors judgment**
1	Selection bias	Sequence generation	Describe the methods used, if any, to generate the allocation sequence in sufficient detail to allow an assessment whether it should produce comparable groups.	Was the allocation sequence adequately generated and applied? (*)
2	Selection bias	Baseline characteristics	Describe all the possible prognostic factors or animal characteristics, if any, that are compared in order to judge whether or not intervention and control groups were similar at the start of the experiment.	Were the groups similar at baseline or were they adjusted for confounders in the analysis?

3	Selection bias	Allocation concealment	Describe the method used to conceal the allocation sequence in sufficient detail to determine whether intervention allocations could have been foreseen before or during enrolment.	Was the allocation adequately concealed? (*)
4	Performance bias	Random housing	Describe all measures used, if any, to house the animals randomly within the animal room.	Were the animals randomly housed during the experiment?
5	Performance bias	Blinding	Describe all measures used, if any, to blind trial caregivers and researchers from knowing which intervention each animal received. Provide any information relating to whether the intended blinding was effective.	Were the caregivers and/or investigators blinded from knowledge which intervention each animal received during the experiment?
6	Detection bias	Random outcome assessment	Describe whether or not animals were selected at random for outcome assessment, and which methods to select the animals, if any, were used.	Were animals selected at random for outcome assessment?
7	Detection bias	Blinding	Describe all measures used, if any, to blind outcome assessors from knowing which intervention each animal received. Provide any information relating to whether the intended blinding was effective.	Was the outcome assessor blinded?
8	Attrition bias	Incomplete outcome data	Describe the completeness of outcome data for each main outcome, including attrition and exclusions from the analysis. State whether attrition and exclusions were reported, the numbers in each intervention group (compared with total randomized animals), reasons for attrition or exclusions, and any re-inclusions in analyses for the review.	Were incomplete outcome data adequately addressed? (*)
9	Reporting bias	Selective outcome reporting	State how selective outcome reporting was examined and what was found.	Are reports of the study free of selective outcome reporting? (*)
10	Other	Other sources of bias	State any important concerns about bias not covered by other domains in the tool.	Was the study apparently free of other problems that could result in high risk of bias? (*)

* Items in agreement with the items in the Cochrane Risk of Bias tool.

Table S5. Modified SYRCLE's RoB tool for assessing risk of bias in *in vitro* research studies.

Item	Type of bias	Domain	Description of domain	Review authors judgment
1	Selection bias	Sample size calculation	Details of the sample size calculation method (software, formula and parameters used to calculate the sample size) should be clearly indicated.	Was the sample size properly calculated and applied?
2	Selection bias	Baseline characteristics	Details of cell lines used.	Is it clear whether the cell lines used are primary cell cultures or continuous cell lines from the gastrointestinal system?
3	Performance bias	Detailed explanation of intervention	The intervention procedure must be clearly indicated, indicating the concentration, the vehicle and the incubation time of the substances administered.	Were interventions administered to cells in a consistent and standardized manner throughout the experiment?
4	Performance bias	Detailed explanation of culture conditions	Provide any information relating to culture conditions.	Are culture conditions comparable between different studies or groups within studies?
5	Detection bias	Details of comparison group	Details of comparison group (positive control, negative control, or standard) should be clearly specified.	Were appropriate controls included?
6	Detection bias	Method of measurement of outcome	The procedure and rationale for the choice of method, and how the results were evaluated should be clearly stated.	Were the statistical analysis clearly specified?
7	Detection bias	Blinding	Details on all measures used, if any, to prevent outcome assessors from knowing which intervention each cell culture received. Provide any information regarding the effectiveness of the planned blinding.	Was the outcome assessor blinded? (*)
8	Attrition bias	Incomplete outcome data	Describe the completeness of outcome data for each main outcome, including attrition and exclusions from the analysis.	Were incomplete outcome data adequately addressed? (*)
9	Reporting bias	Selective outcome reporting	State how selective outcome reporting was examined and what was found.	Are reports of the study free of selective outcome reporting? (*)
10	Other	Other sources of bias	State any important concerns about bias not covered by other domains in the tool.	Was the study apparently free of other problems that could result in high risk of bias? (*)

* Items consistent with the items in the SYRCLE tool.
Each item was rated as "Yes" (low risk of bias), "No" (high risk of bias) or "Unclear" (insufficient information).
For animal studies, Item 1 was considered fulfilled if the study reported at least three technical replicates and three independent biological replicates.

Table S6. Bibliographic details of excluded studies.

Author/Year	Title
Ambroszkiewicz et al, 2024	Comparative Analysis of Myokines and Bone Metabolism Markers in Prepubertal Vegetarian and Omnivorous Children
Chen et al., 2020	Irisin promotes osteogenic differentiation of bone marrow mesenchymal stem cells by activating autophagy via the Wnt//β-catenin signal pathway
Ewendt et al., 2021	Myostatin regulates the production of fibroblast growth factor 23 (FGF23) in UMR106 osteoblast–like cells
Hatakeyama et al., 2025	Exercise-induced interactions between skeletal muscle and bone via myokines and osteokine in mice: Role of FNDC5/irisin, IGF-1, and osteocalcin
Kim et al., 2023	Aortic carboxypeptidase-like protein, a putative myokine, stimulates the differentiation and survival of bone-forming osteoblasts
Li et al., 2025	The effect of rabbit skeletal muscle on the large segment autologous bone ectopic preconstruction of vascularized bone flap
Liu et al., 2025	Skeletal muscle-derived IL-33 mediates muscle-to-bone crosstalk and regulates bone metabolism via CD8+ T cell-secreted CCL5
Otero-Tarrazón et al., 2023	Muscle regeneration in gilthead sea bream: Implications of endocrine and local regulatory factors and the crosstalk with bone
Shanazari et al., 2021	MyomiR-OsteomiR crosstalk induced by different modes and intensities of exercise training and its role in controlling osteogenic differentiation in old male Wistar rats
Shi et al., 2022	Exercised accelerated the production of muscle-derived kynurenic acid in skeletal muscle and alleviated postmenopausal osteoporosis through the Gpr35/NFκB p65 pathway
Storlino et al., 2020	Irisin Prevents Disuse-Induced Osteocyte Apoptosis
Varela-Eirín et al., 2022	Extracellular vesicles enriched in connexin 43 promote a senescent phenotype in bone and synovial cells contributing to osteoarthritis progression
Yu et al., 2021	Bone Marrow Homeostasis Is Impaired via JAK/STAT and Glucocorticoid Signaling in Cancer Cachexia Model

Table S7. Overview of the literature involving animal and cellular studies.

Author/Year	Species/Cells	Sample size	Experimental groups	Type of intervention[a]	Main outcome[b]
M. Chen et al., 2024 [39]	C57BL/6J mice	N/A	Mice:<br>- sham/OVX<br>- TSA-EVs OVX/UN-EVs OVX/OVX with PBS<br>- WT-EV/KD-EV/OE-EV/TSA-WT-EV/TSA-KD-EV	EVs	Histone deacetylase inhibition has a protective effect on osteoporosis in mice by stimulating the release of EVs from HSMM.<br>TSA-EVs promote osteogenic differentiation of hBMSCs.
	HSMM, hBMSCs	None	Cells:<br>- control/UN-EVs/TSA-EVs<br>- HSMM-EV/TSA-HSMM-EV/Non-Ex-EV/Exercise-EV		
Tu-Shen Chen et al., 2025 [51]	C2C12, mouse BMSCs lines D1	None	Control/OCP	CM	Myoblast CM incubated with OCP enhanced the osteogenic differentiation of MSCs.
Hoang et al., 2026 [52]	iMSCs, C2C12	None	iMSC-Gel(L)/iMSC-Gel(M)/iMSC-Gel(H)<br>iMSC-Gel(M)/iMSC-Gel(M)-C2C12	CM	iMSC spheroids exposed to the myoblast secretome and cultured in stiffer hydrogels exhibited enhanced osteogenic differentiation.
Lee et al., 2019 [31]	C57BL/6 mice	N/A	Mice:<br>- PBS/myotube CM<br>- OVX + PBS/OVX + myoblast CM/OVX + myotube CM	CM	Myotube CM contributed to bone formation by inhibiting *in vitro* bone resorption and stimulating preosteoblast viability and migration.<br>Systemic treatment with myotube CM significantly increased bone mass in OVX mice.
	C2C12, BMMs, MC3T3-E1, primary mouse OBs	None	Cells:<br>- control/myoblast CM/myotube CM		

Li et al., 2023 [23]	C57BL/6J mice<br>mouse BMSCs, C2C12	24<br>None	Mice:<br>- sham/OVX/OVX + Exo-shNC/OVX + Exo-shPrrx2<br>Cells:<br>- PM/OM/OM + Exo-shNC/OM + Exo-shPrrx2<br>- OM/OM + Exo-shNC/OM + Exo-shPrrx2/OM + Exo-shPrrx2 + vector/OM + Exo-shPrrx2 + MIR22HG<br>- OM + shNC/OM + shMIR22HG/OM + sh-MIR22HG + inhibitor NC/OM + sh-MIR22HG + miR-128 inhibitor<br>- OM + mimics NC/OM + miR-128 mimics/OM + inhibitor NC/OM + miR-128 inhibitor	EVs[c]	Myoblast-derived exosomal Prrx2 enhanced osteogenic differentiation of BMSCs *in vitro* by transcriptional activation of MIR22HG and activating the Hippo pathway via sponging miR-128 to increase YAP1 expression.<br>Myoblast-derived exosomal Prrx2 diminished bone loss in OVX mice.
Ma et al., 2023 [36]	C57BL/6J mice<br>MC3T3-E1, C2C12, mouse BMSCs	N/A<br>None	Mice:<br>- vehicle (PBS)/2-DG/TE + PBS/TE + 2-DG<br>- vehicle / BoNT/A + vehicle / BoNT/A + Mu-EVs / BoNT/A + Mu-EVs + 2-DG<br>- vehicle/AAV-sh-NC/AAV-sh-Ldha<br>Cells:<br>- vehicle (PBS)/Mu-EVs	EVs	Skeletal muscle secretes EVs that promote osteogenic differentiation of BMSCs and protect against disuse osteoporosis in mice.

Saiz et al., 2019 [32]	SD rats<br>human MSCs, MC3T3-E1, rBMSCs, L6 rat myoblasts	N/A<br>None	Rats:<br>- gels containing SPH-MYO<br>- rBMSC-loaded gels<br>- gels containing SPH-MYO and rBMSCs<br>- empty gels<br>Cells:<br>- MONO<br>- SPH<br>- SPH-MYO	CM	Myokines secreted by L6 myoblasts, previously exposed to the secretome of MSC spheroids, induce osteogenic differentiation *in vitro* and promote bone healing *in vivo*.
Takafuji et al., 2021 [20]	C2C12, bone marrow cells, primary mouse OBs, MC3T3-E1, ST-2, RAW 264.7	None	Transfection of MC3T3, ST-2 and RAW 264.7 cells with miRNA negative control/miRNA mimic MC3T3-E1 treated with Myo-EV/MC3T3-E1 treated without Myo-EV	EVs	Myoblast secreted microRNA-196a-5p suppresses osteoclast-like cell formation and mitochondrial energy metabolism in mouse cells.
Takafuji et al., 2020 [44]	C2C12, NIH3T3, RAW 264.7, mouse bone marrow cells, primary mouse OBs	None	OC precursors treated with Myo-EVs from C2C12 myoblasts/OC precursors treated without Myo-EVs from C2C12 myoblasts<br>Primary OBs treated with Myo-EVs from C2C12 myoblasts/primary OBs treated without Myo-EVs from C2C12 myoblasts<br>Raw 264.7 cells treated without Myo-EVs/Raw 264.7 cells treated with Myo-EVs from C2C12 myoblasts/Raw 264.7 cells treated with Myo-EVs from C2C12 myotubes/Raw 264.7 cells treated with Myo-EVs from NIH3T3	EVs	OC formation in mouse bone marrow cells and Raw 264.7 cells is suppressed by EVs secreted from C2C12 cells.
Takafuji et al., 2021 [45]	C2C12, mouse bone marrow cells, RAW 264.7, primary mouse OBs	None	Myo-EV/FFSS-Myo-EV	Myo-EV, FFSS Myo-EV	Exposure of C2C12 cells to FFSS enhances the suppressive effects of muscle cell-derived EVs on OC formation, while no effect was observed on the phenotype of primary mouse OBs.

Takafuji et al., 2023 [37]	C57BL/6J mice<br>C2C12, ST-2	N/A<br>None	Mice:<br>- control + saline/STZ + saline/control + Myo-EVs/STZ + Myo-EVs<br>- hydrogel with saline/hydrogel with Myo-EVs<br>Cells:<br>- control/BMP-2/BMP-2 + Myo-EVs/BMP-2 + AGE3/BMP-2 + Myo-EVs + AGE3/BMP-2 + high glucose/BMP-2 + Myo-EVs + high glucose	EVs	Administration of muscle-derived EVs enhances osteoblastic differentiation in diabetic mice and thereby improves delayed bone repair.
Tao et al., 2024 [40]	C57BL/6J mice<br>C2C12, MC3T3-E1	N/A<br>None	Mice:<br>- sham/OVX/OVX + treadmill<br>- sham/OVX/OVX + recombinant irisin<br>Cells:<br>- ctrl/CM from myotubes/ov-FNDC5-CM	CM and EVs	Irisin-enriched EVs (exosomes), derived from myotube and induced by exercise, have a protective effects on bone.<br>*In vitro* irisin promotes the differentiation of OBs.
Xing et al., 2024 [41]	SD rats<br>primary mouse skeletal muscle cells, MC3T3-E1	24<br>None	Rats:<br>- sham/DN/DN+ES<br>- sham/DN/DN+ES-Exo<br>Cells:<br>- sham-Exo/DN-Exo/DN+ES-Exo	EVs[c]	Skeletal muscle-derived EVs (exosomes) may promote osteogenesis *in vivo* and *in vitro*.
Xu et al., 2023 [16]	C57BL/6J mice<br>hBMSCs, C2C12	60<br>None	Mice:<br>- PBS/Dex/Dex + Exosome/Dex + Exosome + miR-92a-3p agomir/Dex + Exosome-MS/Dex + Exosome-MS + miR-92a-3p antagomir<br>Cells:<br>- control/Dex/Dex + Exo/Dex + Exo-MS<br>- NC/miRNA mimics/miRNA inhibitor	EVs[c]	EVs (exosomes) derived from mechanical stress-induced myoblasts had a positive impact on the proliferation and differentiation of BMSCs through miR-92a-3p/PTEN/Akt signaling pathway.<br>miR-92a-3p mimics reverse dexamethasone-induced osteoporosis *in vitro* and *in vivo*.

Xu et al., 2018 [43]	C2C12, MC3T3-E1	None	Mock/Myo-Exo/miR-27a-3p-deprived Myo-Exo	EVs[c]	C2C12 myoblast-derived EVs (exosomes) promote OB differentiation of MC3T3-E1 cells through miR-27a-3p component.
W. Chen et al., 2024 [19]	ICR, SAMP6, SAMR1	12 (ICR) + 30 (SAMP6) + 6 (SAMR1)	Mice:<br>- control ICR model (saline) + ICR + ZGF<br>- control SAMP6 model (saline)/SAMP6 + ZGF low dose/SAMP6 + ZGF medium dose/SAMP6 + ZGF high dose/SAMP6 + CaCO3 + alpha D3/negative control (SAMR1 model + saline)	EVs[c]	ZGF can promote osteogenic differentiation of BMSCs through myoblast and myoblast-derived exosomal miRNA.
	mouse BMSCs, C2C12	None	Cells:<br>- control/inducer/inducer + C2C12/inducer + blank-C2C12/inducer + ZGF-C2C12<br>- control/inducer/inducer + Exo/inducer + blank-Exo/inducer + ZGF-Exo		ZGF has a potential therapeutic role in senile osteoporosis *in vivo* through upregulating miR-5100, miR-450b-5p and miR-126a-3p or downregulating miR-669a-5p.
Huang et al., 2023 [35]	C57BL/6J mice	50	Mice:<br>- WT/BTX/SNX<br>- WT/BTX/SNX/WT + Mu-EVs/BTX + Mu-EVs/SNX + Mu-EVs<br>- sham (different as WT) + saline/BTX + saline/SNX + saline/sham + Mu-EVs/BTX + Mu-EVs/SNX + Mu-EVs	Healthy vs. atrophic muscle EVs	Muscle-derived EVs from normal skeletal muscle promote the osteogenic differentiation of BMSCs and inhibit the OC formation *in vitro*. *In vivo* Mu-EVs enhance bone formation and suppress bone resorption in disuse-induced osteoporosis after BTX and SNX treatment.
	mouse BMSCs, primary mouse OBs	None	Cells:<br>- saline/Mu-EVs		In contrast, muscle-derived EVs from atrophic skeletal muscles attenuate the osteogenesis of BMSCs and strengthen the osteoclastogenesis of monocytes.

Amore et al., 2024 [38]	Mice (unknown strain)<br>C2C12, MC3T3	6<br>None	Mice:<br>- sedentary/trained<br>Cells:<br>- vehicle/rec-Gal-3/Gal-3 Ab<br>- CM from EV-transfected C2C12 /CM from FLAG-Gal-3 transfected C2C12<br>- untreated/CM from unstretched C2C12/CM from stretched C2C12/CM from stretched C2C12 + Gal-3 Ab	CM	Gal-3 is significantly increased in the type IIb fibers of trained mice compared to sedentary mice.<br>Gal-3 is released during myogenic differentiation and mechanical stimulation, impairing the early phases of osteogenesis by effects on Notch and Akt activity.
Magarò et al., 2021 [34]	C57BL/6 mice<br>C2C12, 2T3, C57	18 (molecular biology assays and histology) + 8 (vivo gene transfer)<br>None	Mice:<br>- control (nonelectroporated)/empty vector/SOST vector<br>Cells:<br>- CM from C2C12 on day 0 of differentiation (reference)/CM from C2C12 on day 1 of differentiation/CM from C2C12 on day 6 of differentiation/CM from C2C12 on day 14 of differentiation	CM	Myogenic medium inhibited bone cell differentiation *in vitro*. They hypothesized a possible role of sclerostin.<br>Muscular sclerostin has an inhibitory role on bones *in vivo*.
Andrew Norton et al., 2022 [5]	C57BL/6J mice<br>primary mouse OCs, mouse BMSCs	N/A<br>None	Mice:<br>- sham/OVX<br>- ERα-WT/ERα-cKO<br>Cells:<br>- no media/control/muscle CM<br>- control/muscle CM from sham-operated mice/muscle CM from OVX mice<br>- control/muscle CM from ERα-WT mice/muscle CM from ERα-cKO mice	CM	Muscle CM from OVX or skeletal muscle deficient in ERα expression enhances OC differentiation and activity.<br>Mineralization of the mouse BMSCs from the ERα-cKO was not significantly different from the ERα-WT.

Takada et al., 2022 [46]	C2C12, mouse bone marrow cells, RAW 264.7, ST-2	None	MB-EVs/MB-TNF-EVs/MT-EVs/MT-TNF-EVs	EVs	Stimulation of C2C12 muscle cells with TNF-α blunts the OC formation suppression and the osteoblastic differentiation promotion.
B. Wang et al., 2025 [18]	Patients, SD rats<br>C2C12, C3H10 T1/2	10 (for patients); N/A (for rats)<br>None	Rats:<br>- control (removal of adipose tissue around the ovaries)/OVX (removal of bilateral ovaries)<br>- muscle atrophy (injection of BTXA)/control (injection of physiological saline)<br>Cells:<br>- exosomes from control myotubes (treated with DMSO)/exosomes from atrophic myotubes (treated with pyrvinium pamoate)<br>- control coculture C2C12 and C3H10 T1/2 (DMSO group)/coculture C2C12 and C3H10 T1/2 with GW4869 (GW4869 group)	EVs[c]	Atrophic muscle-derived EVs (exosomes) inhibit osteogenic differentiation *in vitro* and *in vivo*.
Y. Wang et al., 2025 [42]	C57BL/6 mice<br>MC3T3-E1, HEK293, C2C12, primary mouse myoblasts	7 (4 male and 3 female)<br>None	Mice:<br>- WT/Slo1 CKO<br>- EXO-shNC/EXO-shSlo1<br>Cells:<br>- control/EXO-shNC/EXO-shSlo1<br>- mimic-NC/mimic-miR-222-3p/inhibitor-NC/inhibitor-miR-222-3p	EVs[c]	Slo1 deficiency in muscle results in decreased bone mass *in vivo* and inhibited osteogenic differentiation *in vitro*.

Xiong et al., 2020 [33]	Lmna$^{f/f}$ mice, Lmna^{HSA}-cko mice, Lmna^{Ocn}-cko mice<br>———<br>mouse BMSCs, primary mouse BMMs, C2C12	N/A<br>———<br>None	Mice:<br>- Lmna$^{f/f}$/Lmna^{HSA}-cko<br>- Lmna$^{f/f}$/Lmna^{Ocn}-cko<br>- HSA-Cre/Lmna$^{f/f}$/Lmna^{HSA}-cko<br>- Lmna$^{f/f}$/Lmna^{HSA}-cko/IL-6-KO/IL-6-KO; Lmna ^{HSA}-cko<br>———<br>Cells:<br>- control myotube CM/Lmna-KO myotube CM<br>- control myotube CM/Lmna-KO myotube CM/Lmna-KO myotube CM + IL-6 Ab	CM	*In vivo* loss of lamin A/C in skeletal muscles results in not only muscle-aging-like deficit but also trabecular bone loss.<br>CM from Lmna-KO myotubes increased osteoclastogenesis *in vitro*.
Chen et al., 2025 [50]	C2C12, MC3T3-E1	None	C2C12/GO-treated C2C12	EVs[c]	EVs (exosomes) derived from GO-treated C2C12 myoblasts upregulated genes such as PDGFRB, COL12A1 and TBX2, while downregulated inflammation-related genes such as C3.
Fernandes et al., 2024 [22]	HCAEC (a_EC), HUVEC (v_EC), human AoSMC (VSMC), primary human OBs	None	Ctrl/O.M./v_EC CM/a_EC CM/VSMC CM	CM	Vascular smooth muscle cell-derived EVs (exosomes) promote the transition of OBs to osteocytes via the β-catenin signaling pathway.
Fernandes et al., 2026 [53]	HCAEC, HUVEC, AoSMC (VSMC), primary human OBs, human MSCs	None	Ctrl/O.M./AoSMC CM/d-AoSMC CM<br>Ctrl/O.M./AoSMC EVs/d-AoSMC EVs	CM and EVs	VSMC, especially under quiescent conditions, promotes osteoblast-to-osteocyte transition.

Lara-Castillo et al., 2023 [21]	TOPGAL mice<br>MLO-Y4, NIH3T3, C2C12	10<br>None	Mice:<br>- PBS + loading/Botox + loading<br>Cells:<br>- Ringer solution/Sol-CM noncontracted/Sol-CM contracted/EDL-CM noncontracted/EDL-CM contracted<br>- blank/NIH3T3-CM/MB-CM/MT-CM<br>- blank static/MB-CM static/MT-CM static/blank + FFSS/MB-CM + FFSS/MT-CM + FFSS	CM	Muscle contraction *in vivo* produces factors that condition or facilitate the osteocyte's ability to respond to mechanical loading.<br>Muscle cells secrete factors that activate PI3K/Akt and β-catenin signaling pathways *in vitro* and *ex vivo*.
Lunde et al., 2023 [47]	Human satellite cells, hBMSCs	None	Control/OB-CM<br>Control/SKM-CM	CM	Inkubation of hBMSCs (differentiated to osteoblastic cells) with SKM-CM resulted in increased glucose uptake and reduced oleic acid uptake. Similar effects were observed after treatment of human skeletal muscle cells with OB-CM.
Suresh Kumar et al., 2023 [48]	C2C12, MC3T3	None	Control/END/HIIT	CM	In the END regimen, ALP, an early osteogenic marker, was significantly upregulated, whereas in the HIIT regimen, intermediate and late-stage osteogenic markers, Col1a1 and Spp1, were significantly upregulated under hyperglycemic conditions.
Yin et al., 2024 [49]	Primary human myocytes, human bone marrow cells, human peripheral blood monocytes	6 (for patients)<br>None	PBS/BAG6^{low} Exos/BAG6^{hi} Exos	EVs[c]	Serum exosomal BAG6 levels and BMD are negatively correlated, indicating their role in synchronized muscle and bone aging.

Abbreviations: HSMM: human skeletal muscle myoblasts; hBMSCs: human bone marrow mesenchymal stem/stromal cells; N/A: not applicable; OVX: ovariectomized; TSA-EVs: EVs isolated from Trichostatin A treated myoblast differentiation CM; UN-EVs: EVs isolated from untreated myoblast differentiation CM; WT: wildtype; KD: knockdown; OE: overexpression; Non-ex-EV: EVs isolated from nonexercising OVX mice; Exercise-EV: EVs isolated from exercising OVX mice; EV(s): extracellular vesicle(s); CM: conditioned medium; C2C12: mouse muscle cells; BMSCs: bone marrow MSCs; OCP: octacalcium phosphate; EGFP: enhanced green fluorescent protein; AER: aerobic exercise; RES: resistance exercise; HA/FNDC5: HA-tagged FNDC5 (N-terminal); IGF-1/HA: HA-tagged IGF-1 (C-terminal); OCN: osteocalcin; iMSCs: induced pluripotent stem cell-derived MSCs; iMSC-Gel(L): iMSCs sferoid-encapsulated gelatin hydrogels with

low stiffness; iMSC-Gel(M): iMSCs sferoid-encapsulated gelatin hydrogels with moderate stiffness; iMSC-Gel(H): iMSCs sferoid-encapsulated gelatin hydrogels with high stiffness; iMSC-Gel(M)-C2C12: iMSC-Gel(M) samples cocultured with C2C12 cells; BMMs: bone marrow macrophages/monocytes; MC3T3-E1: murine preosteoblasts; Exo-shNC: exosomes derived from myogenic differentiated C2C12 cells transfected with negative control shRNA; Exo-shPrrx2: exosomes derived from myogenic differentiated C2C12 cells transfected with shRNA targeting Prrx-2; PM: proliferation medium; OM: osteoblastic medium; MIR22HG: lncRNA-MIR22 host gene; shNC: negative control shRNA; shMIR22HG: shRNA targeting MIR22HG; NC: negative control; 2-DG: 2-deoxy-D-glucose; TE: treadmill exercise; BoNT/A: botulinum neurotoxin type A; Mu-EVs: EVs from skeletal muscle; AAV: adenoassociated viral vector; AAV-sh-NC: AAV negative control Mu-EVs; Ldha: lactate dehydrogenase A; AAV-sh-Ldha: AAV vector expressing shRNA targeting Ldha; SD: Sprague-Dawley; MSCs: mesenchymal stem/stromal cells; rBMSCs: rat bone marrow stromal cells; SPH-MYO: spheroid-myoblast-conditioned media; MONO: MSC monolayer-conditioned media; SPH: spheroid conditioned media; OBs: osteoblasts; OCs: osteoclasts; RAW 264.7: mouse monocytic cells; Kat: kynurenine aminotransferase; Kyna: kynurenic acid; CID-2745687: GPR35 antagonist; END: endurance; HIIT: high-intensity interval training; ST-2: mouse mesenchymal stromal cells; Myo-EV: EVs from mouse muscle C2C12 cells; NIH3T3: mouse fibroblastic cells; FFSS: fluid flow shear stress; FFSS-Myo-EV: EVs secreted from C2C12 cells exposed with FFSS; STZ: streptozotocin; BMP-2: bone morphogenetic proteins-2; AGE3: advanced glycation end product 3; ov-FNDC5-CM: CM from myotubes, transfected with FNDC5 plasmid; DN: denervation group; DN+ES: denervation + electrical stimulation group; Dex: dexamethasone; Exosome/Exo: exosomes derived from unmechanically stimulated myocytes (C2C12); Exosome-MS/Exo-MS: exosomes derived from mechanically stimulated myocytes (C2C12); Exo: exosomes; Myo-Exo: myoblasts-derived exosomes; ICR: outbred mice, derived from Swiss albino mice; SAMP6: senescence-accelerated mouse prone 6 strain of mice; SAMR1: senescence-accelerated mouse resistant mice; ZGF: Zhuang-Gu-Fang; inducer: osteogenic induction medium; inducer + C2C12: inducer, C2C12 myoblasts; inducer + blank-C2C12: inducer, C2C12 myoblasts treated with blank serum; inducer + ZGF-C2C12: inducer, C2C12 myoblasts treated with ZGF serum; inducer + Exo: exosomes, osteogenic induction medium; inducer + blank-Exo group: exosomes after blank serum intervention, osteogenic induction medium; inducer + ZGF-Exo: exosomes after ZGF serum intervention, osteogenic induction medium; BTX: botulinum toxin; SNX: sciatic neurotomy; rec-Gal-3: recombinant soluble form of Galectin-3; Ab: antibody; EV-transfected C2C12: empty vector transfected C2C12; FLAG-Gal-3: a mouse FLAG-tagged Gal-3 encoding plasmid; FLAG-Gal-3 transfected C2C12: Flag-Gal-3 OE C2C12; 2T3: osteoblastic cell line; C57: mouse primary myoblasts; ERα: estrogen receptor α; ERα-cKO: skeletal muscle specific knockout of ERα; KO: knockout; MB-EVs: EVs from C2C12 myoblasts; MT-EVs: EVs from C2C12 myotubes; MB-TNF-EVs: EVs derived from C2C12 myoblasts treated with tumor necrosis factor-α; MT-TNF-EVs: EVs derived from C2C12 myotubes treated with tumor necrosis factor-α; C3H10 T1/2: mouse embryonic cell line, exhibiting fibroblast morphology; BTXA: botulinum toxin type A; GW4869: exosome secretion inhibitor; HEK293: human embryonic kidney cells 293; Slo1 CKO: Slo1 conditional knockout mice; EXO-shNC: exosomes derived from myogenic differentiated C2C12 cells transfected with negative control shRNA; EXO-shSlo1: exosomes derived from myogenic differentiated C2C12 cells transfected with shRNA targeting Slo1; Lmna$^{f/f}$: floxed Lmna mice; Lmna^{HSA}-cko: skeletal muscle–specific Lmna conditional knockout mice; Lmna^{Ocn}-cko: OB-selective Lmna conditional knockout mice; HSA: human alpha-skeletal actin; Cre: cyclization recombination enzyme; LMNA: lamin A/C gene; GO: graphene oxide; HCAEC: human coronary artery endothelial cells; HUVEC: human umbilical vein endothelial cells; AoSMCs: aortic smooth muscle cells; d-AoSMC: shear-stressed AoSMCs; VSMC: vascular smooth muscle cells; O.M.: osteogenic medium; MLO-Y4: murine osteocyte-like cells; Sol-CM: CM from Soleus muscle; EDL-CM: CM from Extensor Digitorum Longus muscle; MB-CM: myoblast CM; MT-CM: myotube CM; OB-CM: CM from OBs; SKM-CM: CM from myotubes; MIET; moderate intensity endurance training; HIET; high intensity ET; MIRT: moderate intensity resistance training; HIRT: high intensity RT; BAG6: BCL2-associated athanogene 6; BAG6^{low}-Exos: blood exosomes with higher expression of BAG6 from aged individuals; Bag6^{hi}-Exos: blood exosomes with higher expression of BAG6 from young individuals; BMD: bone mineral density.

[a]CM vs EVs vs exosomes vs myokines.

[b]Green = net anabolic/antiresorptive; red = net catabolic/proresorptive; without colour = other effects.

[c]The authors used "exosomes", relabelled here as EVs, because no biogenesis-specific evidence was provided.
